# Long‐Term Anifrolumab Treatment Normalizes Hematologic Parameters and Several Serologic Markers in Patients With Systemic Lupus Erythematosus

**DOI:** 10.1002/acr2.90065

**Published:** 2026-06-15

**Authors:** Edward M. Vital, Zahir Amoura, Kenneth C. Kalunian, Ian N. Bruce, Yoshiya Tanaka, Susan Manzi, Ihor Hupka, Jacob Knagenhjelm, Hussein Al‐Mossawi, Catharina Lindholm

**Affiliations:** ^1^ Leeds Institute of Rheumatic and Musculoskeletal Medicine, Faculty of Medicine and Health University of Leeds Leeds United Kingdom; ^2^ French National Reference Center for Systemic Lupus Erythematosus Hôpital La Pitié‐Salpêtrière, Sorbonne University Paris France; ^3^ School of Medicine University of California San Diego, La Jolla; ^4^ Centre for Musculoskeletal Research The University of Manchester Manchester United Kingdom; ^5^ Centre for Public Health, Faculty of Medicine, Health and Life Sciences Queen's University Belfast Belfast United Kingdom; ^6^ The First Department of Internal Medicine University of Occupational and Environmental Health Japan Kitakyushu Japan; ^7^ Lupus Center of Excellence, Autoimmunity Institute, Allegheny Health Network Pittsburgh Pennsylvania; ^8^ BioPharmaceuticals Research and Development AstraZeneca Warsaw Poland; ^9^ BioPharmaceuticals Research and Development AstraZeneca Gothenburg Sweden; ^10^ St Edmund Hall, University of Oxford Oxford United Kingdom

## Abstract

**Objective:**

To evaluate the long‐term effects of anifrolumab on hematologic and serologic parameters over four years.

**Methods:**

This analysis included 536 patients with moderate‐to‐severe systemic lupus erythematosus (SLE) who received intravenous anifrolumab 300 mg (n = 358) or placebo (n = 178) in the 52‐week, phase 3 TULIP‐1/2 trials (NCT02446912 and NCT02446899) and continued the same treatment in the 3‐year, long‐term extension (LTE, NCT02794285), or would have done if not discontinued early; 369 patients entered the LTE. Changes from baseline to week 208 in lymphocytes, hemoglobin, platelets, neutrophils, complement C3, C4, anti–double‐stranded DNA (dsDNA), and Igs were analyzed descriptively. British Isles Lupus Assessment Group–based Composite Lupus Assessment (BICLA) response at week 52 was analyzed by treatment and lymphocyte, hemoglobin, and platelet normalization in responders versus nonresponders, regardless of treatment.

**Results:**

Numerically greater improvements from baseline in lymphocyte, hemoglobin, platelet, and neutrophil levels were observed with anifrolumab over placebo. Comparing anifrolumab versus placebo, lymphocyte and hemoglobin normalization rates were higher and platelet normalization was comparable. BICLA response was associated with lymphocyte, hemoglobin, and platelet normalization over four years, regardless of treatment. Conversely, BICLA responses were higher with anifrolumab versus placebo, irrespective of baseline lymphocyte, hemoglobin, and platelet levels. Improvements in anti‐dsDNA, C3, C4, and Igs from baseline were greater with anifrolumab versus placebo.

**Conclusion:**

The normalization of hematologic parameters and sustained improvements in serologic markers support the long‐term efficacy of anifrolumab in patients with moderate‐to‐severe SLE. Clinical response to anifrolumab was associated with improvements in biomarkers, suggesting restoration of overall immune health.

## INTRODUCTION

One of the early manifestations of systemic lupus erythematosus (SLE) is hematologic involvement, which can present before diagnosis per SLE classification criteria, predominates within the first years of the disease, and can persist throughout the disease course.^1^, ^2^, ^3^ The most frequent hematologic abnormalities among patients include but are not limited to anemia (>50% of patients), lymphopenia (20%–75%), thrombocytopenia (mild: 25%–50%; severe: 10%), leukopenia (22%–42%), and neutropenia (20%–40%), which are associated with increased disease activity, infection risk, bleeding risk, and organ damage accrual.^1^, ^4^, ^5^, ^6^, ^7^, ^8^, ^9^ Patients with SLE may also have increased infection risk due to conventional immunosuppressive therapy, which can worsen disease‐associated T‐cell and B‐cell abnormalities, including CD8+ T‐cell exhaustion and impaired cytolytic function.^10^, ^11^ Serologic biomarkers of SLE are linked to disease activity and immune dysregulation, including complement proteins (C3 and C4), Igs (IgG, IgA, and IgM), and autoantibodies (anti–double‐stranded DNA [anti‐dsDNA], anti‐RNP, and anti‐Sjögren syndrome antibodies A/B [anti‐SSA/anti‐SSB]).^12^ There is growing evidence that high type I interferon (IFN) levels are a key driver in the pathogenesis of active SLE, including hematologic abnormalities (lymphopenia, anemia, thrombocytopenia, and neutropenia), low complement levels, and the presence of autoantibodies including anti‐dsDNA, anti‐SSA, and anti‐RNP.^13^, ^14^, ^15^, ^16^

Anifrolumab, a fully human IgGκ monoclonal antibody that inhibits the type I IFN receptor, is a biologic therapy approved in multiple countries for the treatment of patients with moderate‐to‐severe SLE in addition to standard therapy.^17^, ^18^, ^19^, ^20^, ^21^ The phase 2 MUSE trial, three‐year MUSE open‐label extension trial, and phase 3, placebo‐controlled TULIP‐1/2, and long‐term extension (LTE) trials demonstrated the safety and efficacy of anifrolumab, with reduced disease activity and glucocorticoid (GC) use over a period of up to four years.^22^, ^23^, ^24^, ^25^, ^26^ A post hoc analysis of the MUSE trial showed that one year of anifrolumab treatment reversed SLE‐related lymphopenia, neutropenia, and thrombocytopenia,^27^ and an LTE analysis demonstrated greater improvements in hematologic and serologic measures with anifrolumab versus placebo.^28^ Because hematologic abnormalities in SLE can persist for many years,^1^ we aimed to evaluate the long‐term impact of anifrolumab compared with placebo on hematologic and serologic parameters over the four‐year TULIP plus LTE period.

## MATERIALS AND METHODS

### Study design and patients

Full details on the phase 3, randomized, placebo‐controlled, double‐blind TULIP‐l (NCT02446912), TULIP‐2 (NCT02446899), and LTE (NCT02794285) trials including the study designs, methods, procedures, and inclusion/exclusion criteria are previously described.^22^, ^23^, ^24^ Briefly, eligible patients were adults (aged 18–70 years) with moderate‐to‐severe SLE receiving standard therapy who fulfilled the 1997 American College of Rheumatology classification criteria at TULIP‐1/2 entry.^23^, ^24^, ^25^, ^26^, ^27^, ^28^, ^29^ During the LTE, investigators were permitted to add or modify standard therapy, such as tapering of immunosuppressants and GCs, which was encouraged to reflect real‐world practice but not required.^22^

Here, hematologic and serologic changes were analyzed in patients who were randomized to receive intravenous anifrolumab 300 mg or placebo in the TULIP‐1/2 trials and who continued with the same treatment in the LTE period, or would have continued if not discontinued early (the combined TULIP + LTE population).^22^ Data for patients randomized to placebo in TULIP‐1/2 who were rerandomized to anifrolumab 300 mg in the LTE were not included in this analysis. For key hematologic and serologic assessments, the subgroup of patients who were randomized to anifrolumab 300 mg or placebo in TULIP‐1/2, continued in the LTE, and received the same treatment in TULIP‐1/2 and the LTE (the LTE population) was also analyzed.^22^ The combined TULIP + LTE population and the LTE population are identical after LTE‐study entry (week 52).

This study was conducted in accordance with principles of the Declaration of Helsinki and the International Conference on Harmonisation Guidance for Good Clinical Practice. All patients provided informed consent, and the study was approved by the ethics committee or institutional review board.

### Data collection

Baseline was defined as the last measurement before randomization and investigational product administration on day 1 of the TULIP‐1/2 trials. Hematologic and serologic assessments were analyzed from blood samples collected at baseline, every four weeks during the TULIP study period until week 52 (end of TULIP/LTE‐entry),^23^, ^24^ and during the LTE period at select time points up to week 208 (end of LTE).^22^

### Hematology

All hematologic parameters (lymphocytes, hemoglobin, platelets, and neutrophils) were measured using complete blood cell counts in the combined TULIP + LTE population and in the LTE population. Mean changes from baseline were assessed in prespecified analyses, and hematologic levels were also stratified post hoc into the following subgroups: (1) low (lymphocytes <1 10^^^9 cells [GI]/L; hemoglobin <120 g/L; platelets <150 GI/L; and neutrophils <1.5 GI/L), (2) normal (lymphocytes ≥1 GI/L and <4 GI/L; platelets ≥150 GI/L and <450 GI/L) or normal/high (hemoglobin ≥120 g/L; neutrophils ≥1.5 GI/L), (3) high (lymphocytes ≥4 GI/L; platelets ≥450 GI/L), or (4) withdrawal.

### British Isles Lupus Assessment Group–based Composite Lupus Assessment response

Normalization of lymphocytes, hemoglobin, and platelets from baseline was analyzed post hoc by the British Isles Lupus Assessment Group (BILAG)–based Composite Lupus Assessment (BICLA) response at week 52 in the combined TULIP + LTE population only, regardless of treatment allocation. BICLA response was defined as all of the following: reduction of any moderate‐to‐severe baseline disease activity as measured by changes in BILAG‐2004 A and B domain scores to B/C/D and C/D, respectively, with no worsening in any of the nine BILAG‐2004 organ systems, no worsening of the SLE Disease Activity Index‐2000 (SLEDAI‐2K) score from baseline, no increase of ≥0.3 points in the Physician Global Assessment (range 0–3) from baseline, no discontinuation of investigational product, and no use of restricted medications beyond protocol‐allowed thresholds.^23^ BICLA response rates at week 52 were also analyzed by the treatment group and by subgroups with low or normal/high lymphocyte, hemoglobin, and platelet levels at baseline.

### Serology

Changes in serologic markers (C3, C4, IgG, IgA, IgM, and anti‐dsDNA) were measured using immunosorbent assays. Mean changes from baseline in C3, C4, and anti‐dsDNA antibody levels were assessed in prespecified analyses in the combined TULIP + LTE population and in the LTE population; mean changes from baseline in IgG, IgA, and IgM levels were analyzed post hoc only in the combined population.

### Statistical analysis

The following data were analyzed using descriptive statistics: (1) baseline SLE‐related treatments and baseline hematologic and serologic assessments, (2) changes from baseline in hematologic measures and serologic markers, and (3) lymphocyte, hemoglobin, and platelet normalization from baseline by the treatment group, and by BICLA response at week 52 irrespective of the treatment group. BICLA response rates at week 52 by the treatment group and by lymphocyte, hemoglobin, and platelet subgroups at baseline (low vs normal or normal/high) were analyzed using a stratified Cochran–Mantel–Haenszel approach, with stratification factors of SLEDAI‐2K score at screening (10 points), week 0 GC dose (10 mg/day prednisone or equivalent), and type I IFN gene signature test results at screening (high vs low). Patients with missing data were included at each time point for changes from baseline in hematologic and serologic measures. Missing values for BICLA response at week 52 were imputed using last observation carried forward (LOCF) unless week 48 data were also missing, in which case nonresponder imputation was applied. Data underlying the findings described in this manuscript may be obtained in accordance with AstraZeneca's data sharing policy described at https://astrazenecagrouptrials.pharmacm.com/ST/Submission/Disclosure. Reuse is permitted only with permission from AstraZeneca.

## RESULTS

### Baseline patient characteristics

A total of 536 patients received anifrolumab 300 mg or placebo in the TULIP‐1/2 studies and continued the same treatment in the LTE, or would have done if they had not discontinued early (anifrolumab 300 mg, n = 358; placebo, n = 178; the combined TULIP + LTE population). Of these patients, 369 entered the LTE (anifrolumab 300 mg, n = 257; placebo, n = 112; the LTE anifrolumab and LTE placebo groups, respectively). Demographics, disease characteristics, and hematologic and serologic assessments were generally balanced between treatment groups at the start of the TULIP studies in the combined TULIP + LTE population (Table 1) and in the LTE population (Supplementary Table S1, which presents hematologic and some serologic markers only, because demographics and clinical characteristics, including levels of anti‐dsDNA, C3, and C4 were previously published for this population^22^).

**Table 1:** SLE‐related treatments, and hematologic and serologic parameters at TULIP baseline in the combined TULIP + LTE groups*

Characteristic	Anifrolumab 300 mg (n = 358)	Placebo (n = 178)
Baseline SLE treatments		
GC^a^		
GC dose,^b^ mg/day		
≤10	169 (47.2)	88 (49.4)
≥10	189 (52.8)	90 (50.6)
Mean (± SD)	9.6 (9.9)	9.9 (8.7)
Immunosuppressants, n (%)		
Yes	172 (48.0)	90 (50.6)
Antimalarials, n (%)		
Yes	242 (67.6)	134 (75.3)
Hematology parameters, n (%) unless otherwise stated		
Lymphocytes		
Mean (SD), GI/L	1.3 (0.7)	1.3 (0.6)
Low (<1 GI/L)	138 (38.5)	74 (41.6)
Normal (≥1 GI/L and <4 GI/L)	219 (61.2)	103 (57.9)
High (≥4 GI/L)	1 (0.3)	1 (0.6)
Hemoglobin		
Mean (SD), g/L	125.1 (14.8)	126.7 (15.8)
Low (<120 g/L)	115 (32.1)	51 (28.7)
Normal/high (≥120 g/L)	243 (67.9)	127 (71.3)
Platelets		
Mean (SD), GI/L	239.9 (78.3)	248.2 (76.5)
Low (<150 GI/L)	38 (10.6)	16 (9.0)
Normal (≥150 and <450 GI/L)	315 (88.0)	160 (89.9)
High (≥4 GI/L)	5 (1.4)	2 (1.1)
Neutrophils		
Mean (SD), GI/L	3.8 (1.8)	4.1 (2.1)
Low (<1.5 GI/L)	1.1 (0.3)	1.1 (0.3)
Normal/high (≥1.5 GI/L)	3.9 (1.8)	4.2 (2.1)
Serology markers		
Anti‐dsDNA positive^c^		
n (%)	165 (46.1)	69 (38.8)
Mean (SD), U/mL	129.6 (262.9)	115.3 (166.4)
Low C3^c^		
n (%)	129 (36.0)	63 (35.4)
Mean (SD), g/L	0.7 (0.2)	0.7 (0.1)
Low C4^c^		
n (%)	83 (23.2)	37 (20.8)
Mean (SD), g/L	0.07 (0.02)	0.07 (0.01)
IgG^d^, mean (SD), g/L	14.3 (5.2)	13.8 (4.8)
IgA^d^, mean (SD), g/L	3.1 (1.7)	3.0 (1.4)
IgM^d^, mean (SD), g/L	1.1 (0.9)	1.0 (0.6)

* C3, complement 3; C4, complement 4; dsDNA, double‐stranded DNA; GC, glucocorticoid; GI, 10^^^9 cells; LTE, long‐term extension; SLE, systemic lupus erythematosus.

^a^ GC contains prednisone dose or equivalent.

^b^ Includes patients not taking GC at the corresponding visit (0 mg/day).

^c^ Includes only patients with baseline positive anti‐dsDNA or abnormal C3 or C4 levels.

^d^ Mean (SD) calculated from n = 354 patients in the anifrolumab group and n = 176 patients in the placebo group.

Among the combined TULIP + LTE population at baseline, thrombocytopenia was present in 11% and 9% of the anifrolumab 300 mg and placebo groups, respectively, and lymphopenia present in 39% and 42%, respectively (Table 1). Low C4 levels (23% and 21%) and positive anti‐dsDNA levels (46% and 39%) were present at baseline.

### Lymphocyte levels over time

**Figure 1:**
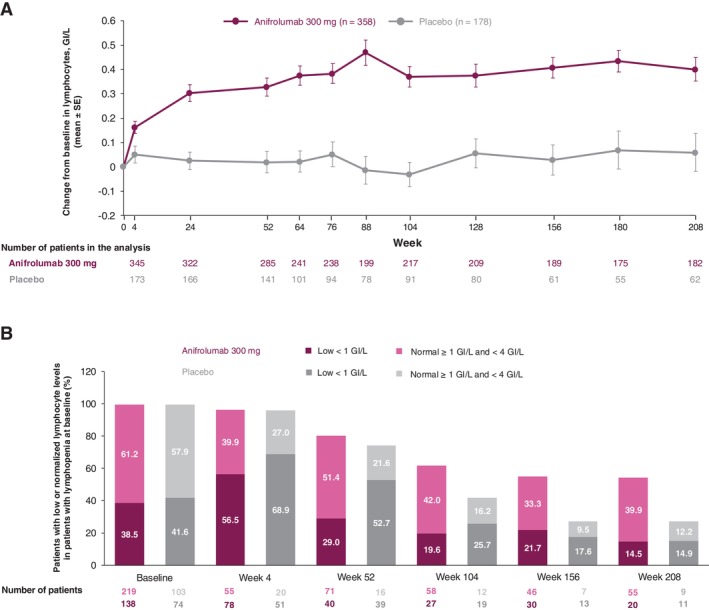
Effect of anifrolumab treatment on lymphocyte levels in the combined TULIP + LTE groups. (A) Change from baseline in lymphocyte levels. (B) Proportions of patients with low or normalized lymphocyte levels over time in patients with lymphopenia at baseline. The proportions of patients in panel B do not add up to 100% due to missing data. Baseline data only show proportions of patients with lymphopenia (low) or normal lymphocyte levels at baseline. Data for patients with high lymphocyte levels are not shown in this analysis. GI, 10^^^9 cells; LTE, long‐term extension.

Overall, numerically greater increases in mean lymphocyte levels were observed in the combined anifrolumab versus the combined placebo group, irrespective of baseline lymphocyte levels (Figure 1A). For patients with low lymphocyte levels at baseline (lymphopenia: <1 GI/L), patients receiving anifrolumab had higher rates of lymphocyte normalization compared with placebo, and this effect was maintained from week 4 (39.9%, n = 55 vs 27.0%, n = 20) through week 208 (39.9%, n = 55 vs 12.2%, n = 9; Figure 1B). The percentage of patients with persistent lymphopenia post‐baseline tended to decrease over time in both treatment groups. Similar trends were observed in the LTE anifrolumab and placebo groups (Supplementary Figure S1). Although the percentage of patients with baseline lymphopenia who withdrew generally increased in both treatment groups over time, withdrawal rates were numerically lower in the anifrolumab group compared with placebo, and the treatment difference was maintained from week 4 (0.7%, n = 1 vs 2.7%, n = 2) to week 208 (35.5%, n = 49 vs 54.1%, n = 40); withdrawal rates increased over time in both treatment groups (Supplementary Figure S2).

### Hemoglobin levels over time

**Figure 2:**
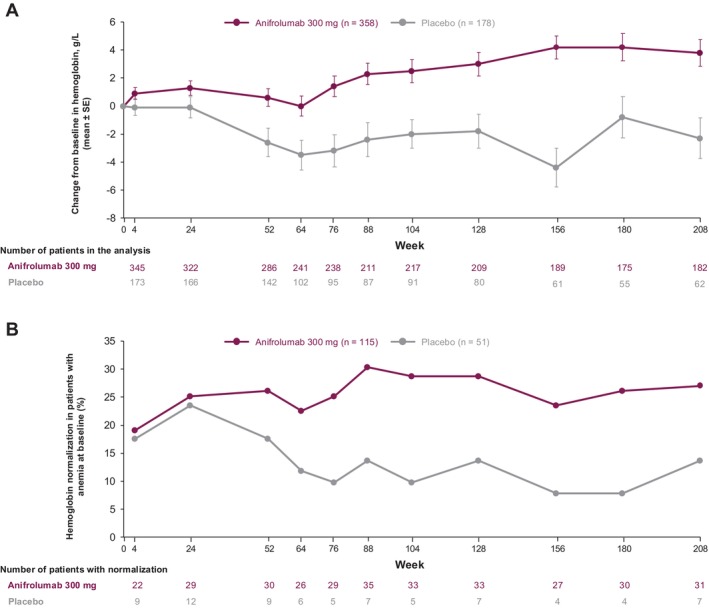
Effect of anifrolumab treatment on hemoglobin levels in the combined TULIP + LTE groups. (A) Change from baseline in hemoglobin levels. (B) Hemoglobin normalization over time in patients with anemia at baseline. LTE, long‐term extension

Numerically greater increases in mean hemoglobin levels were observed with anifrolumab combined group versus placebo combined group, irrespective of baseline levels (Figure 2A). For patients with low hemoglobin levels at baseline (anemia: <120 g/L), a higher proportion of anifrolumab‐treated patients had normal/high hemoglobin levels compared with placebo, and this effect was maintained over time from week 4 (19.1%, n = 22 vs 17.6%, n = 9) to week 208 (27.0%, n = 31 vs 13.7%, n = 7; Figure 2B). Similar trends were observed in the LTE groups (Supplementary Figure S3). Withdrawal rates in patients with anemia were numerically lower in the anifrolumab group compared with placebo from week 40 (12.2%, n = 14 vs 17.6%, n = 9) to week 208 (35.7%, n = 41 vs 54.9%, n = 28), and rates increased over time in both treatment groups (Supplementary Figure S4).

### Platelet levels over time

**Figure 3:**
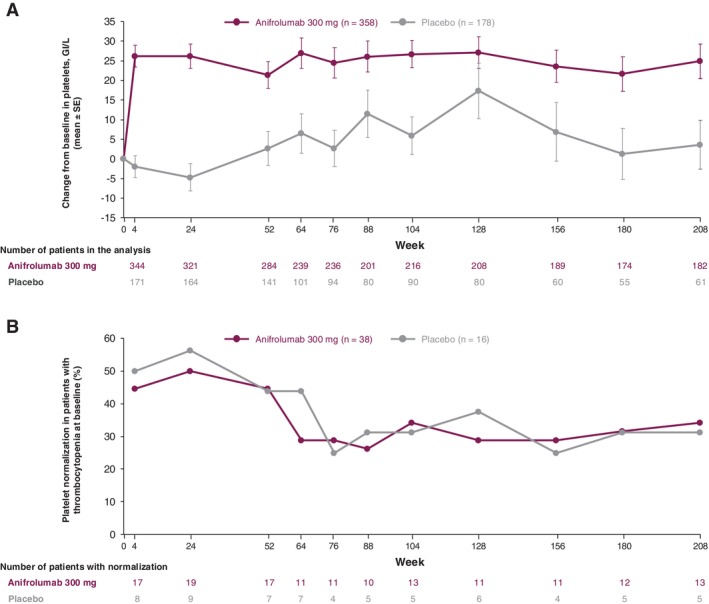
Effect of anifrolumab treatment on platelet levels in the combined TULIP + LTE groups. (A) Change from baseline in platelet levels. (B) Platelet normalization over time in patients with thrombocytopenia at baseline. No increases in mean platelet levels from baseline led to high platelet counts above the normal range at any time point (ie, mean levels did not exceed ≥450 GI/L). GI, 10^^^9 cells; LTE, long‐term extension

Numerically greater increases in mean platelet levels were observed early with anifrolumab versus placebo in the combined groups, irrespective of baseline levels (Figure 3A). For patients with low platelet levels at baseline (thrombocytopenia: <150 GI/L), no clear trends were observed for rates of platelet normalization in the anifrolumab and placebo groups from week 4 (44.7%, n = 17 vs 50.0%, n = 8) to week 208 (34.2%, n = 13 vs 31.3%, n = 5; Figure 3B); similar trends were observed in the LTE groups (Supplementary Figure S5). Withdrawal rates in patients with thrombocytopenia generally increased over time in both treatment groups; withdrawal rates were numerically higher with anifrolumab versus placebo as early as week 8 (2.6%, n = 1 vs 0%) up to week 88 (26.3%, n = 10 vs 18.8%, n = 3) and were numerically lower with anifrolumab versus placebo from week 104 to 208 (Supplementary Figure S6).

### Normalization of lymphocyte, hemoglobin, and platelet levels in BICLA responders versus nonresponders

**Figure 4:**
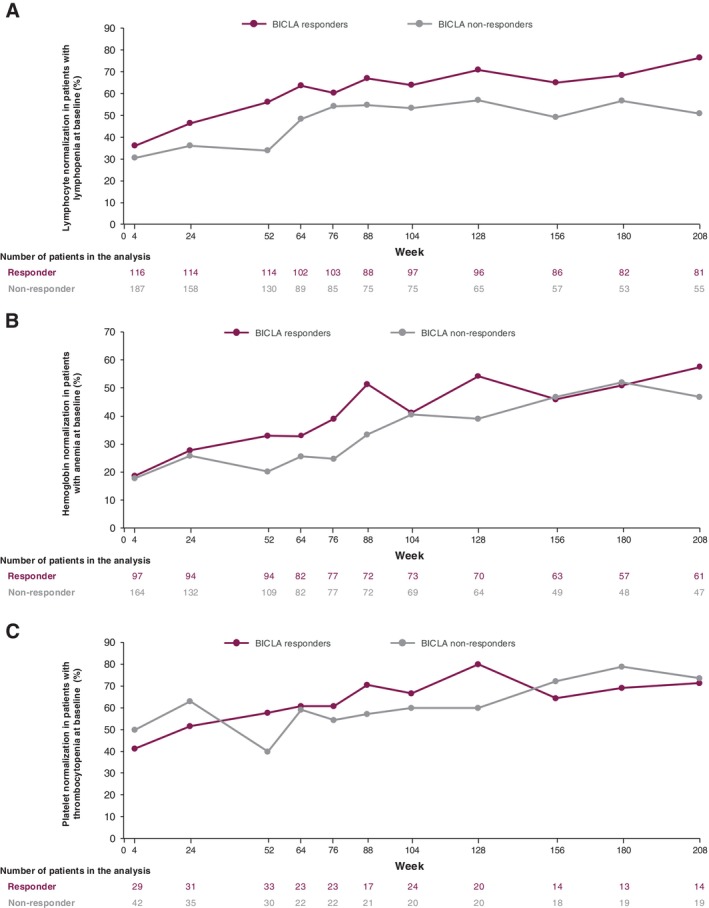
Lymphocyte, hemoglobin, and platelet levels were associated with BICLA response, regardless of treatment: Association between week 52 BICLA response and normalization (low to normal or high) of (A) lymphocytes, (B) hemoglobin, and (C) platelet levels over time, among all patients with low baseline levels in the combined TULIP + LTE groups. BICLA, British Isles Lupus Assessment Group–based Composite Lupus Assessment; LTE, long‐term extension.

Among all of patients with “low” hematologic parameters at baseline, normalization (low to normal or normal/high) of lymphocytes, hemoglobin, and platelet levels at week 52 were associated with BICLA response (responders vs nonresponders, lymphocytes: 56.1% [64 of 114] vs 33.8% [44 of 130]; hemoglobin: 33.0% [31 of 94] vs 20.2% [22 of 109]; platelets: 57.6% [19 of 33] vs 40.0% [12 of 30]). The percentage of patients with lymphocyte normalization from baseline was numerically higher among BICLA responders versus nonresponders at every visit from week 4 (36.2% [42 of 116] vs 30.5% [57 of 187]) to week 208 (76.5% [62 of 81] vs 50.9% [28 of 55]) (Figure 4A). Rates of patients with hemoglobin normalization from baseline were also higher in BICLA responders for most time points from week 4 (18.6% [18 of 97] vs 17.7% [29 of 164]) to week 128 (54.3% [38 of 70] vs 39.1% [25 of 64]) (Figure 4B). Platelet normalization rates from baseline were associated with BICLA response during part of the LTE period from week 52 (57.6% [19 of 33] vs 40.0% [12 of 30]) to week 128 (80.0% [16 of 20] vs 60.0% [12 of 20]) (Figure 4C).

### BICLA response by the treatment group and by lymphocyte, hemoglobin, and platelet levels at baseline

At week 52, BICLA response rates were numerically greater with anifrolumab versus placebo for all patients, irrespective of their hemoglobin, lymphocyte, or platelet levels at baseline. In patients with lymphopenia, anemia, or thrombocytopenia at baseline, percentages of BICLA responders were 49.7% (69 of 138), 51.0% (58 of 115), and 42.1% (16 of 38) in the anifrolumab group versus 23.8% (33 of 138), 24.1% (28 of 114), and 40.6 (13 of 32) in the placebo group, respectively (Supplementary Figure S7). In patients with normal or normal/high lymphocyte, hemoglobin, and platelet levels at baseline, BICLA response rates were 46.4% (102 of 220), 46.7% (113 of 243), and 48.3% (155 of 320) with anifrolumab versus 35.2% (78 of 222), 34.0% (83 of 246), and 30.0% (98 of 328) with placebo, respectively.

### Neutrophil levels over time

Changes in mean neutrophil levels from baseline were numerically greater in the anifrolumab combined group versus placebo combined group irrespective of baseline levels from week 4 (mean ± SD change from baseline: anifrolumab, 0.79 ± 1.86 GI/L; placebo, −0.004 ± 1.74 GI/L) to week 208 (anifrolumab, 0.58 ± 2.37 GI/L; placebo, −0.18 ± 2.15 GI/L; Supplementary Figure S8A). Improvements in patients with low baseline neutrophils (<1.5 GI/L) were greater with anifrolumab versus placebo for most time points and as early as week 4 (mean ± SD change from baseline: anifrolumab, 1.69 ± 1.45 GI/L; placebo, 0.07 ± 0.23 GI/L) through week 64 (anifrolumab, 1.59 ± 0.76 GI/L; placebo, 1.46 ± 1.61 GI/L), and a similar trend was observed from week 104 to 208 (Supplementary Figure S8B). In patients with normal or high baseline neutrophils (≥1.5 GI/L) changes from baseline were greater with anifrolumab versus placebo for all time points as early as week 4 (mean ± SD change from baseline: anifrolumab, 0.76 ± 1.87 GI/L; placebo, −0.006 ± 1.77 GI/L) through week 208 (anifrolumab, 0.54 ± 2.39 GI/L; placebo, −0.24 ± 2.12 GI/L). Similar trends were seen in the LTE groups (Supplementary Figure S9).

### Changes in anti‐dsDNA and complement levels over time

**Figure 5:**
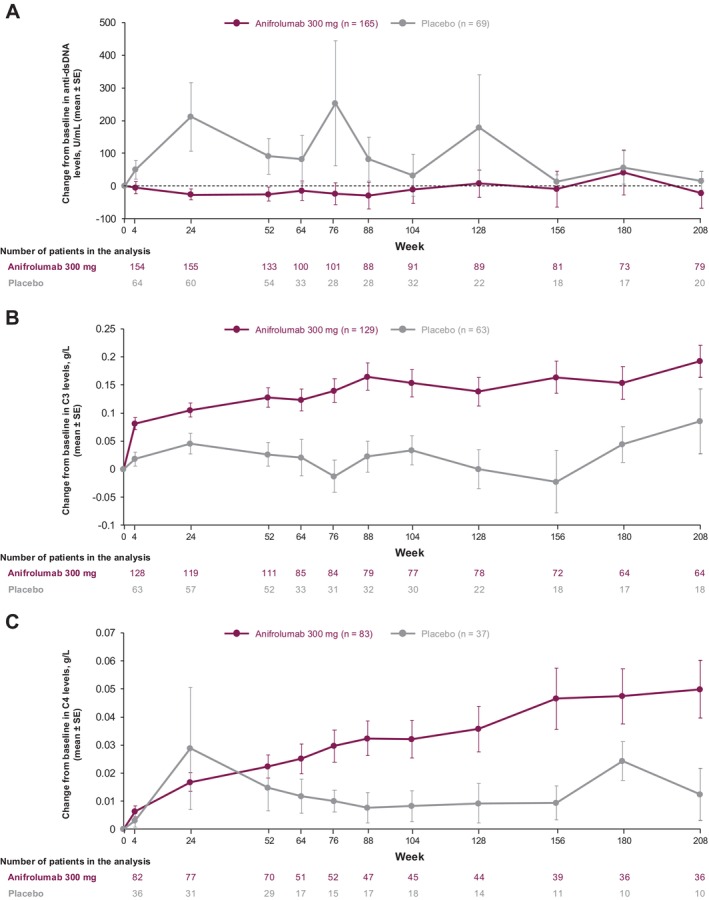
Effects of anifrolumab treatment on serologic markers in the combined TULIP + LTE groups. Change from baseline in levels of (A) anti‐dsDNA, (B) C3, and (C) C4 over time among patients who were anti‐dsDNA positive or had low C3 or C4 at baseline, respectively. C3, complement 3; C4, complement 4; dsDNA, double‐stranded DNA; LTE, long‐term extension.

Numerically greater reductions in mean anti‐dsDNA levels from baseline were observed with anifrolumab versus placebo as early as week 4 (mean ± SE change from baseline: −5.55 ± 18.65 U/mL vs 50.0 ± 28.12 U/mL), and this trend was maintained to week 208 (mean ± SE: anifrolumab, −22.1 ± 45.64 U/mL; placebo, 15.57 ± 28.66 U/mL; Figure 5A) in the combined TULIP + LTE groups. Improvements in mean C3 levels from baseline were greater with anifrolumab versus placebo and were maintained from week 4 (mean ± SE change from baseline: 0.08 ± 0.01 g/L vs 0.02 ± 0.01 g/L) to week 208 (mean ± SE: 0.19 ± 0.03 g/L vs 0.09 ± 0.06 g/L; Figure 5B). Greater improvements in mean C4 levels from baseline were observed with anifrolumab versus placebo for most time points from week 4 (mean ± SE change from baseline: 0.006 ± 0.002 g/L vs 0.003 ± 0.002 g/L) to week 208 (mean ± SE: 0.05 ± 0.01 g/L vs 0.01 ± 0.009 g/L; Figure 5C). Similar trends were seen in the LTE groups (Supplementary Figure S10).

### Changes in Ig levels over time

Numerically greater reductions from baseline in mean IgG and IgA were observed over time with anifrolumab versus placebo and were maintained from week 24 to 208 (IgG mean ± SD change from baseline: −0.84 ± 2.32 g/L vs 0.54 ± 2.16 g/L and −1.14 ± 3.21 g/L vs 0.34 ± 2.61 g/L; IgA mean ± SD change from baseline: −0.14 ± 0.47 g/L vs 0.05 ± 0.48 g/L and −0.26 ± 0.71 g/L vs −0.07 ± 0.69 g/L). Reductions from baseline in mean IgM levels were lower with anifrolumab compared with placebo for all time points up to week 104 (mean ± SD change from baseline: −0.06 ± 0.46 g/L vs −0.009 ± 0.30 g/L).

## DISCUSSION

Complex immune dysregulation perpetuates both disease activity and risk of infection in patients with SLE.^11^ Existing therapies targeting circulating immune cells directly may add to the burden of immune suppression.^1^, ^6^ More effective, well‐tolerated, targeted therapies that maintain disease control over time while improving hematologic and serologic abnormalities are needed. High type I IFN activity in SLE is associated with greater disease activity, hematologic manifestations (lower hemoglobin and lymphocyte levels), and lower complement levels.^14^, ^15^, ^16^ Type I IFNs have been shown to directly impact the metabolic fitness of CD8+ T cells, resulting in reduced cell viability.^30^ Determining whether hematologic involvement is disease related or due to treatment side effects in patients with SLE may be challenging, because standard immunosuppressives and GC therapies have been associated with lymphopenia, anemia, and/or neutropenia.^1^, ^6^ With the exception of GC, concomitant use of standard therapy was generally comparable during the LTE period between the anifrolumab and placebo groups; further information on concomitant medications was previously reported in the LTE study.^22^ Anifrolumab is a first‐in‐class type I IFN receptor inhibitor approved for treatment of moderate‐to‐severe SLE despite standard of care.^17^, ^18^, ^19^, ^20^, ^21^ This study evaluated hematologic and serologic changes over four years with long‐term anifrolumab treatment in patients with moderate‐to‐severe SLE from the phase 3 TULIP‐1/2 and LTE trials.^22^, ^23^, ^24^ Our findings show that, compared with placebo, patients receiving anifrolumab had early and greater improvements in lymphocyte, hemoglobin, platelet, and neutrophil levels, which were sustained over the four‐year TULIP + LTE period. Long‐term data reported here are valuable for physicians treating patients with anifrolumab (in line with current EULAR recommendations^31^) for the management of this chronic, autoimmune disease.

In this study, the baseline rate of lymphopenia was generally consistent with lymphopenia rates (20%–75%) commonly observed in patients with SLE.^1^ However, baseline rates of anemia and thrombocytopenia were all lower than the reported hematologic manifestation rates observed in SLE (anemia: >50%; mild and severe thrombocytopenia: 25%–50% and 10%, respectively).^1^ In our study, higher rates of lymphocyte and hemoglobin normalization were observed over time in anifrolumab‐treated patients compared with placebo; these treatment differences were maintained until the end of the LTE, at year 4. Rates of patients with persistent lymphopenia or anemia who remained in the study tended to decrease over the four‐year TULIP + LTE period in both treatment groups, although a higher dropout rate was observed in patients receiving placebo plus standard therapy. Long‐term improvements in lymphocyte, hemoglobin, and platelet levels from baseline were all numerically greater with anifrolumab compared with placebo and were sustained over four years. These findings are consistent with a MUSE post hoc analysis, which showed that anifrolumab suppressed inflammatory proteins associated with SLE disease activity, and reversed lymphopenia, neutropenia, and thrombocytopenia.^27^ These results supplement the preliminary observations from the TULIP‐LTE analysis by showing long‐term hematologic changes over time, further supporting the hypothesis that blocking type I IFN signaling with anifrolumab normalizes SLE‐related hematologic parameters in patients with SLE.^28^

Although there may be multiple mechanisms for neutropenia in SLE, type I IFNs have been shown to promote cell death by ferroptosis.^32^ Increased neutrophil cell death also occurs via neutrophil extracellular traps (NETs), an interferogenic process, and correlates with disease activity and cardiovascular risk.^33^, ^34^ A study of patients treated with anifrolumab in the MUSE trial showed that one year of anifrolumab treatment significantly reduced levels of NET complex markers that positively correlated with an IFN gene signature, compared with placebo.^33^ Consistent with these findings, a recent TULIP‐1/2 study of the immunomodulatory effects of anifrolumab demonstrated that NETosis pathways in SLE were downregulated with anifrolumab.^35^ Meanwhile, GC therapy is known to cause neutrophilia by increased mobilization of neutrophils from bone marrow into circulation and reduced migration into tissues.^36^ Our study shows that anifrolumab‐treated patients had greater neutrophil improvements in the overall and high neutrophil subgroups compared with placebo, and these improvements were sustained over the entire four years of the TULIP + LTE period. Even though change from baseline values in patients with neutropenia did not increase over time, improvements from baseline with anifrolumab were numerically greater versus placebo for most time points throughout the study duration. Taken together, these results suggest a mechanism by which anifrolumab may reduce neutrophil cell death and may have implications for restoring neutrophil levels, which are often dysregulated in the immunopathology of SLE.

Lymphopenia is a common hallmark of hematologic involvement in patients with SLE that is significantly associated with disease activity, especially when occurring in conjunction with thrombocytopenia, which independently associates with disease activity.^1^, ^4^, ^5^, ^6^ Importantly, the presence of lymphopenia associates with infection risk, and thrombocytopenia associates with risk of bleeding, organ damage, and death.^4^, ^5^, ^7^, ^9^ In our analysis, the rate of BICLA response was associated with lymphocyte, hemoglobin, and platelet normalization over time, regardless of treatment during the four‐year TULIP + LTE period. These interesting findings suggest that achieving a lower disease activity state confers an overall improvement in SLE hematologic manifestations and associated symptoms. To our knowledge, this is the first report showing that, compared with nonresponders, more patients who are BICLA responders can also achieve hematologic improvements regardless of the treatment type and can sustain these benefits of reduced disease activity over time. Conversely, higher rates of BICLA response were associated with anifrolumab treatment versus placebo, irrespective of hematologic involvement at baseline, suggesting anifrolumab efficacy over standard of care alone in reducing disease activity in both subgroups of patients with hematologic involvement and in those with normal hematologic parameters at baseline. This builds on previous efficacy findings from the TULIP‐1 and TULIP‐2 trials, which demonstrated higher BICLA rates with anifrolumab versus placebo in patients with moderate‐to‐severe SLE.^23^, ^24^

Type I IFNs play crucial roles in generation of germinal centers and B‐cell differentiation to plasma cells.^37^ The presence of autoantibodies including anti‐dsDNA, anti‐SSA, anti‐SSB, and anti‐RNP are also linked to increased type I IFN levels in circulation.^14^, ^15^, ^16^ Serologic assessments over time with long‐term anifrolumab treatment revealed that patients had numerically greater anti‐dsDNA, C3, and C4 improvements from baseline compared with placebo, and the treatment benefit was maintained over time. Reductions in IgG over time were greater in patients receiving anifrolumab compared with placebo, and improvements were generally maintained. These inhibitory effects of anifrolumab on IgG levels might suggest an indirect impact on aberrant B‐cell activity, and are supported by recently published transcriptomic data, which showed downregulation of several B‐cell activating proteins associated with SLE during anifrolumab treatment.^35^ These results support previous efficacy findings with anifrolumab on disease activity, which showed greater BICLA response rates in serologic subgroups of patients with active SLE from the TULIP‐1/2 trials,^38^ and are consistent with the immunosuppressive mechanism of action of anifrolumab in reducing type I IFN signaling and inhibiting autoantibody‐producing cells in SLE.^17^, ^39^

Strengths of this study include the study duration, the double‐blinded trial design, and the number of time points to evaluate normalization of baseline hematologic parameters. This is the longest randomized, placebo‐controlled study to date that directly compares hematologic and serologic effects of anifrolumab plus standard therapy versus standard therapy alone. Limitations of this study are the relatively small sample sizes and that this study was not powered for statistical comparison of efficacy of the studied outcomes between treatment groups. Another limitation that should be considered during interpretation of the results from this LTE study is the potential survival bias, because the rates of patients with persistent hematologic manifestations could be expected to decrease over time. No imputation was performed for patients who withdrew, and increasing dropout rates were observed across both treatment groups for patients with baseline lymphopenia, anemia, or thrombocytopenia over the course of the LTE. Higher withdrawal rates were observed in the placebo group compared with anifrolumab for all hematologic measures reported (lymphocytes, hemoglobin, and platelets). Additionally, there were no treatment differences in platelet normalization rates over time between the anifrolumab and placebo groups, which may be due to lower numbers of patients with thrombocytopenia at baseline compared with the respective lymphocyte and hemoglobin analyses over time, so there remains uncertainty over this effect.

Given the early, chronic, and IFN‐related nature of hematologic manifestations in SLE and the need for targeted therapies over the extended disease duration, determining the long‐term effects of anifrolumab on hematology is crucial. The results of this four‐year, long‐term analysis support the preliminary efficacy findings from the phase 2 MUSE trial of anifrolumab (greater SLE Responder Index‐4 rates including GC tapering),^26^ sustained SLEDAI‐2K disease activity improvements in the MUSE open‐label extension study over three years,^25^ and the hematologic and serologic efficacy of anifrolumab in the MUSE post hoc analysis.^27^ This study builds on the foundational evidence of anifrolumab efficacy seen in the previous phase 3 TULIP‐1, TULIP‐2, and TULIP‐LTE trials (greater BICLA response rates, reduced SLEDAI‐2K scores over time, and lower GC use),^22^, ^23^, ^24^ and further expands on the preliminary laboratory marker analysis of the TULIP‐LTE study.^28^ These data provide additional support for the long‐term efficacy of anifrolumab in patients with active SLE,^22^, ^25^, ^40^ as shown by early and sustained improvements in disease activity, hematologic manifestations, and serologic markers over time. The observations here are consistent with earlier experimental data indicating the role of type I IFNs in promoting the hematologic and serologic abnormalities of SLE.^13^, ^14^, ^15^, ^16^ Thus, anifrolumab therapy may reduce pathogenic inflammation while also improving biomarkers that denote overall immune health. These improvements may be relevant to a wider range of other long‐term outcomes important to patients’ health and quality of life.

## AUTHOR CONTRIBUTIONS

All authors contributed to at least one of the following manuscript preparation roles: conceptualization AND/OR methodology, software, investigation, formal analysis, data curation, visualization, and validation AND drafting or reviewing/editing the final draft. As corresponding author, Dr Vital confirms that all authors have provided the final approval of the version to be published, and takes responsibility for the affirmations regarding article submission (eg, not under consideration by another journal), the integrity of the data presented, and the statements regarding compliance with institutional review board/Declaration of Helsinki requirements. All authors contributed to the investigation, formal analysis of the data, and writing, reviewing, and editing of the manuscript.

## ROLE OF THE STUDY SPONSOR

This study and medical writing support was funded by AstraZeneca. AstraZeneca was involved in the study design, data collection, analysis, and interpretation and in the decision to submit the paper for publication. All authors interpreted the data, critically reviewed the manuscript for important intellectual content, approved the final draft, and agreed to its submission.

## Supporting information


**Disclosure Form**:

**Data S1.** Supporting Information.
