# Supplementary material for: Long‐Term Anifrolumab Treatment Normalizes Hematologic Parameters and Several Serologic Markers in Patients With Systemic Lupus Erythematosus
Source: ACR Open Rheumatol. 2026 Jun 15;8(6):e90065. doi: 10.1002/acr2.90065 (PMC13267668; doi:10.1002/acr2.90065)
Supplement: Supplementary file 1 — Disclosure Form: [file ACR2-8-e90065-s001.pdf]

ICMJE DISCLOSURE FORM

**Date:**	9/6/2025
**Your Name:**	Edward Vital
**Manuscript Title:**	"Long-Term Anifrolumab Treatment Normalizes Hematologic Parameters and Several Serologic Markers in Patients With Systemic Lupus Erythematosus".
**Manuscript Number (if known):**	Click or tap here to enter text.

In the interest of transparency, we ask you to disclose all relationships/activities/interests listed below that are related to the content of your manuscript. "Related" means any relation with for-profit or not-for-profit third parties whose interests may be affected by the content of the manuscript. Disclosure represents a commitment to transparency and does not necessarily indicate a bias. If you are in doubt about whether to list a relationship/activity/interest, it is preferable that you do so.

The author's relationships/activities/interests should be defined broadly. For example, if your manuscript pertains to the epidemiology of hypertension, you should declare all relationships with manufacturers of antihypertensive medication, even if that medication is not mentioned in the manuscript.

In item #1 below, report all support for the work reported in this manuscript without time limit. For all other items, the time frame for disclosure is the past 36 months.

		**Name all entities with whom you have this relationship or indicate none (add rows as needed)**	**Specifications/Comments (e.g., if payments were made to you or to your institution)**
**Time frame: Since the initial planning of the work**			
1	All support for the present manuscript (e.g., funding, provision of study materials, medical writing, article processing charges, etc.) **No time limit for this item.**	☒ None	
**Time frame: past 36 months**			
**2**	Grants or contracts from any entity (if not indicated in item #1 above).	☐ **None**	
		Roche	Grant paid to my employer
		AstraZeneca	Grant paid to my employer
**3**	Royalties or licenses	☐ **None**	
		Easy BILAG	Licensing fees paid to my employer

		Name all entities with whom you have this relationship or indicate none (add rows as needed)	Specifications/Comments (e.g., if payments were made to you or to your institution)
**4**	Consulting fees	☐ **None**	
		Novartis	
		UCB	
**5**	Payment or honoraria for lectures, presentations, speakers bureaus, manuscript writing or educational events	☐ **None**	
		AstraZeneca	
		Novartis	
		Otsuka	
**6**	Payment for expert testimony	☐ **None**	
		NICE	Payment for expert opinion on a new therapy
**7**	Support for attending meetings and/or travel	☐ **None**	
		UCB	Travel to EULAR
**8**	Patents planned, issued or pending	☒ **None**	
**9**	Participation on a Data Safety Monitoring Board or Advisory Board	☐ **None**	
		Dianthus	
		Roche	
		Otsuka	
		BMS	
		AbbVie	
		Ventus	
**10**	Leadership or fiduciary role in other board, society, committee or advocacy group, paid or unpaid	☐ **None**	
		SLEURO	General secretary (unpaid)
		BILAG	Chair (unpaid)

		Name all entities with whom you have this relationship or indicate none (add rows as needed)	Specifications/Comments (e.g., if payments were made to you or to your institution)
**11**	Stock or stock options	☒ **None**	
**12**	Receipt of equipment, materials, drugs, medical writing, gifts or other services	☐ **None**	
**13**	Other financial or non-financial interests	☒ **None**	

Please place an "X" next to the following statement to indicate your agreement:

☒ I certify that I have answered every question and have not altered the wording of any of the questions on this form.

ICMJE DISCLOSURE FORM

Date: 6/11/2025

Your Name: AMOURA

Manuscript Title:

Manuscript Number (if known): Click or tap here to enter text.

In the interest of transparency, we ask you to disclose all relationships/activities/interests listed below that are related to the content of your manuscript. "Related" means any relation with for-profit or not-for-profit third parties whose interests may be affected by the content of the manuscript. Disclosure represents a commitment to transparency and does not necessarily indicate a bias. If you are in doubt about whether to list a relationship/activity/interest, it is preferable that you do so.

The author's relationships/activities/interests should be defined broadly. For example, if your manuscript pertains to the epidemiology of hypertension, you should declare all relationships with manufacturers of antihypertensive medication, even if that medication is not mentioned in the manuscript.

In item #1 below, report all support for the work reported in this manuscript without time limit. For all other items, the time frame for disclosure is the past 36 months.

		**Name all entities with whom you have this relationship or indicate none (add rows as needed)**	**Specifications/Comments (e.g., if payments were made to you or to your institution)**
Time frame: Since the initial planning of the work			
1	All support for the present manuscript (e.g., funding, provision of study materials, medical writing, article processing charges, etc.) **No time limit for this item.**	☒ None	
			Click the tab key to add additional rows.
Time frame: past 36 months			
**2**	Grants or contracts from any entity (if not indicated in item #1 above).	☒ **None**	
**3**	Royalties or licenses	☐ **None**	

		Name all entities with whom you have this relationship or indicate none (add rows as needed)	Specifications/Comments (e.g., if payments were made to you or to your institution)
**4**	Consulting fees	☐ **None**	
		AMGEN, ROCHE, ASTRAZENECA, GSK, NOVARTIS	
**5**	Payment or honoraria for lectures, presentations, speakers bureaus, manuscript writing or educational events	☐ **None**	
		AMGEN, ROCHE, ASTRAZENECA, GSK, NOVARTIS	
**6**	Payment for expert testimony	☒ **None**	
**7**	Support for attending meetings and/or travel	☒ **None**	
**8**	Patents planned, issued or pending	☒ **None**	
**9**	Participation on a Data Safety Monitoring Board or Advisory Board	☒ **None**	
**10**	Leadership or fiduciary role in other board, society, committee or advocacy group, paid or unpaid	☒ **None**	

		Name all entities with whom you have this relationship or indicate none (add rows as needed)	Specifications/Comments (e.g., if payments were made to you or to your institution)
**11**	Stock or stock options	☒ **None**	
**12**	Receipt of equipment, materials, drugs, medical writing, gifts or other services	☒ **None**	
**13**	Other financial or non-financial interests	☒ **None**	

Please place an "X" next to the following statement to indicate your agreement:

☒ I certify that I have answered every question and have not altered the wording of any of the questions on this form.

ICMJE DISCLOSURE FORM

Date: 8/2/2024

Your Name: Kenneth C Kalunian

Manuscript Title: **The Impact of Long-Term Anifrolumab Treatment on Patient-reported Outcomes During a Randomized, Placebo-Controlled Phase 3 Long-Term Extension Trial**

Manuscript Number (if known): Click or tap here to enter text.

In the interest of transparency, we ask you to disclose all relationships/activities/interests listed below that are related to the content of your manuscript. "Related" means any relation with for-profit or not-for-profit third parties whose interests may be affected by the content of the manuscript. Disclosure represents a commitment to transparency and does not necessarily indicate a bias. If you are in doubt about whether to list a relationship/activity/interest, it is preferable that you do so.

The author's relationships/activities/interests should be defined broadly. For example, if your manuscript pertains to the epidemiology of hypertension, you should declare all relationships with manufacturers of antihypertensive medication, even if that medication is not mentioned in the manuscript.

In item #1 below, report all support for the work reported in this manuscript without time limit. For all other items, the time frame for disclosure is the past 36 months.

		**Name all entities with whom you have this relationship or indicate none (add rows as needed)**	**Specifications/Comments (e.g., if payments were made to you or to your institution)**
		Time frame: Since the initial planning of the work	
1	All support for the present manuscript (e.g., funding, provision of study materials, medical writing, article processing charges, etc.) **No time limit for this item.**	☒ **None**	
			Click the tab key to add additional rows.
		Time frame: past 36 months	
2	Grants or contracts from any entity (if not indicated in item #1 above).	☒ **None**	
3	Royalties or licenses	☒ **None**	

		Name all entities with whom you have this relationship or indicate none (add rows as needed)	Specifications/Comments (e.g., if payments were made to you or to your institution)
4	Consulting fees	☐ None	
		AstraZeneca	
5	Payment or honoraria for lectures, presentations, speakers bureaus, manuscript writing or educational events	☒ None	
6	Payment for expert testimony	☒ None	
7	Support for attending meetings and/or travel	☐ None	
8	Patents planned, issued or pending	☐ None	
9	Participation on a Data Safety Monitoring Board or Advisory Board	☒ None	
10	Leadership or fiduciary role in other board, society, committee or advocacy group, paid or unpaid	☒ None	

		Name all entities with whom you have this relationship or indicate none (add rows as needed)	Specifications/Comments (e.g., if payments were made to you or to your institution)
11	Stock or stock options	☒ None	
12	Receipt of equipment, materials, drugs, medical writing, gifts or other services	☒ None	
13	Other financial or non-financial interests	☒ None	

Please place an "X" next to the following statement to indicate your agreement:

☒ I certify that I have answered every question and have not altered the wording of any of the questions on this form.

ICMJE DISCLOSURE FORM

Date:______14th June 2025_____________________ ____
Your Name:_______Ian N Bruce MD FRCP____
Manuscript Title: Long-Term Anifrolumab Treatment Normalizes Hematologic Parameters and Several Serologic Markers in Patients With Systemic Lupus Erythematosus.
Manuscript number (if known):____

In the interest of transparency, we ask you to disclose all relationships/activities/interests listed below that are related to the content of your manuscript. "Related" means any relation with for-profit or not-for-profit third parties whose interests may be affected by the content of the manuscript. Disclosure represents a commitment to transparency and does not necessarily indicate a bias. If you are in doubt about whether to list a relationship/activity/interest, it is preferable that you do so.

The following questions apply to the author's relationships/activities/interests as they relate to the current manuscript only.

The author's relationships/activities/interests should be defined broadly. For example, if your manuscript pertains to the epidemiology of hypertension, you should declare all relationships with manufacturers of antihypertensive medication, even if that medication is not mentioned in the manuscript.

In item #1 below, report all support for the work reported in this manuscript without time limit. For all other items, the time frame for disclosure is the past 36 months.

		**Name all entities with whom you have this relationship or indicate none (add rows as needed)**	**Specifications/Comments (e.g., if payments were made to you or to your institution)**
		**Time frame: Since the initial planning of the work**	
1	All support for the present manuscript (e.g., funding, provision of study materials, medical writing, article processing charges, etc.) **No time limit for this item.**		
		**Time frame: past 36 months**	
2	Grants or contracts from any entity (if not indicated in item #1 above).	Astra Zeneca	Grant support to institution
		Janssen	Grant support to institution
		Otsuka	Grant support to institution
		GSK	Grant support to institution
		Novartis	Grant support to institution
3	Royalties or licenses	____None	

4	Consulting fees	BMS	Consulting fees to institution
		Astra Zeneca	Consulting fees to institution
		GSK	Consulting fees to institution
		Janssen	Consulting fees to institution
		Cabaletta Bio	Consulting fees to institution
		Lilly	Consulting fees to institution
		UCB	Consulting fees to institution
5	Payment or honoraria for lectures, presentations, speakers bureaus, manuscript writing or educational events	Novartis	Speaker Fees to Institution
		GSK	Speaker Fees to Institution
		Astra Zeneca	Speaker Fees to Institution
		Janssen	Speaker Fees to Institution
6	Payment for expert testimony	____None	
7	Support for attending meetings and/or travel	____None	
8	Patents planned, issued or pending	____None	
9	Participation on a Data Safety Monitoring Board or Advisory Board	GSK	Fees to institution
		Astra Zeneca	Fees to institution
10	Leadership or fiduciary role in other board, society, committee or advocacy group, paid or unpaid	____None	
11	Stock or stock options	____None	
12	Receipt of equipment, materials, drugs, medical writing, gifts or other services	____None	
13	Other financial or non-financial interests	____None	

Please place an "X" next to the following statement to indicate your agreement:

X___ I certify that I have answered every question and have not altered the wording of any of the questions on this form.

ICMJE DISCLOSURE FORM

**Date:**	8/26/2021
**Your Name:**	Yoshiya Tanaka
**Manuscript Title:**	Click or tap here to enter text.
**Manuscript Number (if known):**	Click or tap here to enter text.

In the interest of transparency, we ask you to disclose all relationships/activities/interests listed below that are related to the content of your manuscript. "Related" means any relation with for-profit or not-for-profit third parties whose interests may be affected by the content of the manuscript. Disclosure represents a commitment to transparency and does not necessarily indicate a bias. If you are in doubt about whether to list a relationship/activity/interest, it is preferable that you do so.

The author's relationships/activities/interests should be defined broadly. For example, if your manuscript pertains to the epidemiology of hypertension, you should declare all relationships with manufacturers of antihypertensive medication, even if that medication is not mentioned in the manuscript.

In item #1 below, report all support for the work reported in this manuscript without time limit. For all other items, the time frame for disclosure is the past 36 months.

		**Name all entities with whom you have this relationship or indicate none (add rows as needed)**	**Specifications/Comments (e.g., if payments were made to you or to your institution)**
**Time frame: Since the initial planning of the work**			
1	All support for the present manuscript (e.g., funding, provision of study materials, medical writing, article processing charges, etc.) **No time limit for this item.**	☒ None	
			Click the tab key to add additional rows.
**Time frame: past 36 months**			
**2**	Grants or contracts from any entity (if not indicated in item #1 above).	☒ **None**	
**3**	Royalties or licenses	☒ **None**	

		Name all entities with whom you have this relationship or indicate none (add rows as needed)	Specifications/Comments (e.g., if payments were made to you or to your institution)
**4**	Consulting fees	☒ **None**	
**5**	Payment or honoraria for lectures, presentations, speakers bureaus, manuscript writing or educational events	☐ **None**	
		Chugai, UCB, Abbvie, AstraZeneca, Eli-Lilly, Behringer-Ingelheim, GlaxoSmithKline, Eisai, IQVIA, Daiichi-Sankyo, Otsuka, Taisho, Gilead, Bristol-Mayers	speaking fees and/or honoraria
**6**	Payment for expert testimony	☐ **None**	
**7**	Support for attending meetings and/or travel	☒ **None**	
**8**	Patents planned, issued or pending	☒ **None**	
**9**	Participation on a Data Safety Monitoring Board or Advisory Board	☒ **None**	
**10**	Leadership or fiduciary role in other board, society, committee or advocacy group, paid or unpaid	☒ **None**	

		Name all entities with whom you have this relationship or indicate none (add rows as needed)	Specifications/Comments (e.g., if payments were made to you or to your institution)
**11**	Stock or stock options	☒ **None**	
**12**	Receipt of equipment, materials, drugs, medical writing, gifts or other services	☒ **None**	
**13**	Other financial or non-financial interests	☒ **None**	

Please place an "X" next to the following statement to indicate your agreement:

☒ I certify that I have answered every question and have not altered the wording of any of the questions on this form.

ICMJE DISCLOSURE FORM

**Date:**	6/6/2025
**Your Name:**	Susan Manzi, MD MPH
**Manuscript Title:**	Long-Term Anifrolumab Treatment Normalizes Hematologic Parameters and Several Serologic Markers in Patients With Systemic Lupus Erythematosus
**Manuscript Number (if known):**	Click or tap here to enter text.

In the interest of transparency, we ask you to disclose all relationships/activities/interests listed below that are related to the content of your manuscript. "Related" means any relation with for-profit or not-for-profit third parties whose interests may be affected by the content of the manuscript. Disclosure represents a commitment to transparency and does not necessarily indicate a bias. If you are in doubt about whether to list a relationship/activity/interest, it is preferable that you do so.

The author's relationships/activities/interests should be defined broadly. For example, if your manuscript pertains to the epidemiology of hypertension, you should declare all relationships with manufacturers of antihypertensive medication, even if that medication is not mentioned in the manuscript.

In item #1 below, report all support for the work reported in this manuscript without time limit. For all other items, the time frame for disclosure is the past 36 months.

		**Name all entities with whom you have this relationship or indicate none (add rows as needed)**	**Specifications/Comments (e.g., if payments were made to you or to your institution)**
Time frame: Since the initial planning of the work			
1	All support for the present manuscript (e.g., funding, provision of study materials, medical writing, article processing charges, etc.) **No time limit for this item.**	☒ None	
Time frame: past 36 months			
**2**	Grants or contracts from any entity (if not indicated in item #1 above).	☐ **None**	
		AbbVie	Cartesian Therapeutics
		Amgen	GlaxoSmithKline (GSK)
		AstraZeneca	Human Genome Sciences
		Bristo Myers Squibb (BMS)	

		Name all entities with whom you have this relationship or indicate none (add rows as needed)	Specifications/Comments (e.g., if payments were made to you or to your institution)
3	Royalties or licenses	☒ None	
4	Consulting fees	☐ None	
		SEE ATTACHED TWO PAGES	Susan Manzi
5	Payment or honoraria for lectures, presentations, speakers bureaus, manuscript writing or educational events	☒ None	
6	Payment for expert testimony	☒ None	
7	Support for attending meetings and/or travel	☒ None	
8	Patents planned, issued or pending	☐ None	
		SEE ATTACHED TWO PAGES	Susan Manzi; UPMC; AHN
9	Participation on a Data Safety Monitoring Board or Advisory Board	☐ None	
		DSMB - NIAID	Susan Manzi
		Miltenyi	Susan Manzi
10	Leadership or fiduciary role in	☐ None	

		Name all entities with whom you have this relationship or indicate none (add rows as needed)	Specifications/Comments (e.g., if payments were made to you or to your institution)
	other board, society, committee or advocacy group, paid or unpaid	CAQH – Provider Advisory Board	Susan Manzi
		Lupus Foundation of America – Past Chair/Member	
**11**	Stock or stock options	☒ **None**	
**12**	Receipt of equipment, materials, drugs, medical writing, gifts or other services	☒ **None**	
**13**	Other financial or non-financial interests	☒ **None**	

Please place an "X" next to the following statement to indicate your agreement:

☒ I certify that I have answered every question and have not altered the wording of any of the questions on this form.

DISCLOSURES - SUSAN MANZI, MD, MPH

AstraZeneca	Consultant
Cartesian	Advisory Consultant
CAQH	Provider Advisory Council
Exagen Diagnostics, Inc	Consultant/Patent/Royalties
GSK	Consultant
Lilly	Consultant
Lupus Foundation of America	Past Chair/Member
UCB Advisory Board	Consultant

Current Grant Support

Source/ Grant Number	Title	Role in Project	Years Inclusive
Sponsor: HGS/GSK IRB #: PRO00034756	A 5-Year Prospective Observational Registry to Assess Adverse Events of Interest and Effectiveness in Adults with Active, Autoantibody-Positive Systemic Lupus Erythematosus Treated with or without BENLYSTA™ (belimumab) (SABLE)	Site Principal Investigator	2014-present
Sponsor: Amgen IRB# Pro00072377	A Randomized, Double-blind, Placebo-controlled Phase 4 Clinical Trial to Evaluate the Long-term Safety and Efficacy of Avacopan in Subjects With Antineutrophil Cytoplasmic Antibody (ANCA)-associated Vasculitis (Tavneos Safety)	Co-Investigator	2023-present
Sponsor: Cartesian IRB# DC08-SLE-001	Descartes-08 for patients with systemic lupus erythematosus	Co-Investigator	2024-present
Sponsor: BMS IRB# Pro00066328	A Phase 3, Randomized, Double-blind, Placebo-controlled Study to Evaluate the Efficacy and Safety of Deucravacitinib in Participants with Active Systemic Lupus Erythematosus (POETYK SLE-2)	Co-Investigator	2024-present

Intellectual Property

Patents Examined and Allowed for Issuance as a Patent

Title: Identification of Apolipoprotein H Mutations and Their Diagnostic Uses
US Patent No.: 6,203,980
Issued: March 20, 2001
Inventors: M. Ilyas Kamboh, Dharambir K. Sanghera, and Susan Manzi
Assignees: University of Pittsburgh

Title: Reticulocytes Bearing C4-derived Ligands May Serve as "Instant Messengers" of Disease Activity in Systemic Lupus Erythematosus
Issued: 2008
Inventors: Joseph M. Ahearn, MD and Susan Manzi, MD, MPH
Assignee: University of Pittsburgh
Title: Real Time Method of Detecting Acute Inflammatory Conditions
US Patent No: 7,361,517
Issued: April 22, 2008
Inventors: Joseph M. Ahearn and Susan M. Manzi
Assignee: University of Pittsburgh

Title: Diagnosis and Monitoring of Systemic Lupus Erythematosus and of Scleroderma
US Patent No.: 7,390,631
Issued: June 24, 2008
Inventors: Joseph M. Ahearn and Susan Manzi
Assignee: University of Pittsburgh

Title: Diagnosing and Monitoring Inflammatory Diseases By Measuring Complement Components On White Blood Cells.
US Patent No.:7,585,640
Issued: September 8, 2009
Inventors: Joseph M. Ahearn, Susan Manzi and Chau-Ching Liu
Assignee: University of Pittsburgh

Title:	Cell-Bound Complement Activation Products as Diagnostic Biomarkers for Pre-Lupus
US Patent:	No.: 9,495,517
Issued:	November 15, 2016
Inventors:	Joseph M. Ahearn, Susan Manzi and Chau-Ching Liu
Assignee:	Allegheny Singer Research Institute

Patents Pending

Title:	Anti-Lymphocyte Autoantibodies as Diagnostic Biomarkers
Application Number:	PCT/US15/44372
Inventors:	Joseph M. Ahearn, Susan Manzi and Chau-Ching Liu
Assignee:	Allegheny Singer Research Institute

Title:	Cell-Bound Complement Activation Products Assays as Companion Diagnostics Antibody-Based Drugs
Application Number:	62/199,713
Inventors:	Joseph M. Ahearn, Susan Manzi and Chau-Ching Liu
Assignee:	Allegheny Singer Research Institute

Title:	C4 Gene Copy Number and Cell-Bound Complement Activation Products as Companion Biomarkers for Diagnosis, Monitoring and Stratification of Lupus and Pre-Lupus
Inventors:	Joseph M. Ahearn, Susan Manzi and Chau-Ching Liu
Assignee:	Allegheny Singer Research Institute

Title:	Identification and Monitoring of Systemic Lupus Erythematosus
Atty. Docket	No.: 030182P
Filed:	April 16, 2003
Inventors:	Joseph M. Ahearn and Susan Manzi
Assignee:	University of Pittsburgh

Title:	Monitoring Immunologic, Hematologic and Inflammatory Diseases
Atty. Docket	No.: 030356P
Filed:	June 15, 2003
Inventors:	Joseph M. Ahearn and Susan Manzi
Assignee:	University of Pittsburgh

Title:	Assessing Risk of Cerebrovascular Thrombosis by Measuring C4d on Platelets
Filed:	September 14, 2005
Inventors:	Joseph M. Ahearn and Susan Manzi and Amy Kao
Assignee:	University of Pittsburgh

Title:	Platelet CB-CAPS for Monitoring Inflammation
Filed:	October 2005
Inventors:	Joseph M. Ahearn and Susan Manzi
Assignee:	University of Pittsburgh

ICMJE DISCLOSURE FORM

**Date:**	5/30/2025
**Your Name:**	Ihor Hupka
**Manuscript Title:**	"Long-Term Anifrolumab Treatment Normalizes Hematologic Parameters and Several Serologic Markers in Patients With Systemic Lupus Erythematosus".
**Manuscript Number (if known):**	Click or tap here to enter text.

In the interest of transparency, we ask you to disclose all relationships/activities/interests listed below that are related to the content of your manuscript. "Related" means any relation with for-profit or not-for-profit third parties whose interests may be affected by the content of the manuscript. Disclosure represents a commitment to transparency and does not necessarily indicate a bias. If you are in doubt about whether to list a relationship/activity/interest, it is preferable that you do so.

The author's relationships/activities/interests should be defined broadly. For example, if your manuscript pertains to the epidemiology of hypertension, you should declare all relationships with manufacturers of antihypertensive medication, even if that medication is not mentioned in the manuscript.

In item #1 below, report all support for the work reported in this manuscript without time limit. For all other items, the time frame for disclosure is the past 36 months.

		Name all entities with whom you have this relationship or indicate none (add rows as needed)	Specifications/Comments (e.g., if payments were made to you or to your institution)
**Time frame: Since the initial planning of the work**			
1	All support for the present manuscript (e.g., funding, provision of study materials, medical writing, article processing charges, etc.) **No time limit for this item.**	☒ None	
			I was regular AZ employer at time of development this manuscript
			Click the tab key to add additional rows.
**Time frame: past 36 months**			
**2**	Grants or contracts from any entity (if not indicated in item #1 above).	☒ **None**	
**3**	Royalties or licenses	☒ **None**	

		Name all entities with whom you have this relationship or indicate none (add rows as needed)	Specifications/Comments (e.g., if payments were made to you or to your institution)
**4**	Consulting fees	☒ **None**	
**5**	Payment or honoraria for lectures, presentations, speakers bureaus, manuscript writing or educational events	☒ **None**	
**6**	Payment for expert testimony	☒ **None**	
**7**	Support for attending meetings and/or travel	☒ **None**	
**8**	Patents planned, issued or pending	☒ **None**	
**9**	Participation on a Data Safety Monitoring Board or Advisory Board	☒ **None**	
**10**	Leadership or fiduciary role in other board, society, committee or advocacy group, paid or unpaid	☒ **None**	

		Name all entities with whom you have this relationship or indicate none (add rows as needed)	Specifications/Comments (e.g., if payments were made to you or to your institution)
**11**	Stock or stock options	☒ **None**	
**12**	Receipt of equipment, materials, drugs, medical writing, gifts or other services	☐ **None**	
**13**	Other financial or non-financial interests	☒ **None**	

Please place an "X" next to the following statement to indicate your agreement:

☒ I certify that I have answered every question and have not altered the wording of any of the questions on this form.

ICMJE DISCLOSURE FORM

Date: 6/5/2025

Your Name: Jacob Knagenhjelm

Manuscript Title: Click or tap here to enter text.

Manuscript Number (if known): Click or tap here to enter text.

In the interest of transparency, we ask you to disclose all relationships/activities/interests listed below that are related to the content of your manuscript. "Related" means any relation with for-profit or not-for-profit third parties whose interests may be affected by the content of the manuscript. Disclosure represents a commitment to transparency and does not necessarily indicate a bias. If you are in doubt about whether to list a relationship/activity/interest, it is preferable that you do so.

The author's relationships/activities/interests should be defined broadly. For example, if your manuscript pertains to the epidemiology of hypertension, you should declare all relationships with manufacturers of antihypertensive medication, even if that medication is not mentioned in the manuscript.

In item #1 below, report all support for the work reported in this manuscript without time limit. For all other items, the time frame for disclosure is the past 36 months.

		**Name all entities with whom you have this relationship or indicate none (add rows as needed)**	**Specifications/Comments (e.g., if payments were made to you or to your institution)**
Time frame: Since the initial planning of the work			
1	All support for the present manuscript (e.g., funding, provision of study materials, medical writing, article processing charges, etc.) **No time limit for this item.**	☐ None	
		AstraZeneca	Employee and indirect stockholder through mutual fund
			Click the tab key to add additional rows.
Time frame: past 36 months			
**2**	Grants or contracts from any entity (if not indicated in item #1 above).	☒ **None**	
**3**	Royalties or licenses	☒ **None**	

		Name all entities with whom you have this relationship or indicate none (add rows as needed)	Specifications/Comments (e.g., if payments were made to you or to your institution)
**4**	Consulting fees	☒ **None**	
**5**	Payment or honoraria for lectures, presentations, speakers bureaus, manuscript writing or educational events	☒ **None**	
**6**	Payment for expert testimony	☒ **None**	
**7**	Support for attending meetings and/or travel	☒ **None**	
**8**	Patents planned, issued or pending	☒ **None**	
**9**	Participation on a Data Safety Monitoring Board or Advisory Board	☒ **None**	
**10**	Leadership or fiduciary role in other board, society, committee or advocacy group, paid or unpaid	☒ **None**	

		Name all entities with whom you have this relationship or indicate none (add rows as needed)	Specifications/Comments (e.g., if payments were made to you or to your institution)
**11**	Stock or stock options	☐ **None**	
		AstraZeneca	Indirect stock holder through mutual fund
**12**	Receipt of equipment, materials, drugs, medical writing, gifts or other services	☐ **None**	
**13**	Other financial or non-financial interests	☐ **None**	

Please place an "X" next to the following statement to indicate your agreement:

☒ I certify that I have answered every question and have not altered the wording of any of the questions on this form.

ICMJE DISCLOSURE FORM

Date: 12/1/2024

Your Name: Hussein Al-Mossawi

Manuscript Title: Untargeted Metabolomic Profiling of Patients with Lupus Nephritis Reveals Unique Metabolites that are Modulated Through Type I Interferon Inhibition by Anifrolumab Treatment in a Phase 2 Trial

Manuscript Number (if known): Click or tap here to enter text.

In the interest of transparency, we ask you to disclose all relationships/activities/interests listed below that are related to the content of your manuscript. "Related" means any relation with for-profit or not-for-profit third parties whose interests may be affected by the content of the manuscript. Disclosure represents a commitment to transparency and does not necessarily indicate a bias. If you are in doubt about whether to list a relationship/activity/interest, it is preferable that you do so.

The author's relationships/activities/interests should be defined broadly. For example, if your manuscript pertains to the epidemiology of hypertension, you should declare all relationships with manufacturers of antihypertensive medication, even if that medication is not mentioned in the manuscript.

In item #1 below, report all support for the work reported in this manuscript without time limit. For all other items, the time frame for disclosure is the past 36 months.

		Name all entities with whom you have this relationship or indicate none (add rows as needed)	Specifications/Comments (e.g., if payments were made to you or to your institution)
**Time frame: Since the initial planning of the work**			
1	All support for the present manuscript (e.g., funding, provision of study materials, medical writing, article processing charges, etc.) **No time limit for this item.**	☐ None	
		Former Employee of AstraZeneca	
			Click the tab key to add additional rows.
**Time frame: past 36 months**			
2	Grants or contracts from any entity (if not indicated in item #1 above).	☒ **None**	
3	Royalties or licenses	☒ **None**	

		Name all entities with whom you have this relationship or indicate none (add rows as needed)	Specifications/Comments (e.g., if payments were made to you or to your institution)
**4**	Consulting fees	☒ **None**	
**5**	Payment or honoraria for lectures, presentations, speakers bureaus, manuscript writing or educational events	☒ **None**	
**6**	Payment for expert testimony	☒ **None**	
**7**	Support for attending meetings and/or travel	☒ **None**	
**8**	Patents planned, issued or pending	☒ **None**	
**9**	Participation on a Data Safety Monitoring Board or Advisory Board	☒ **None**	
**10**	Leadership or fiduciary role in other board, society, committee or advocacy group, paid or unpaid	☐ **None**	
		Immunocore	Leadership position
		Oak Hill Bio Let	Non executive director

		Name all entities with whom you have this relationship or indicate none (add rows as needed)	Specifications/Comments (e.g., if payments were made to you or to your institution)
**11**	Stock or stock options	☒ **None**	
		Immunocore	Options
		Oak Hill Bio	Stock
		GSK	Stock
**12**	Receipt of equipment, materials, drugs, medical writing, gifts or other services	☒ **None**	
**13**	Other financial or non-financial interests	☒ **None**	

Please place an "X" next to the following statement to indicate your agreement:

☒ I certify that I have answered every question and have not altered the wording of any of the questions on this form.

ICMJE DISCLOSURE FORM

Date: 6/2/2025

Your Name: Catharina Lindholm

Manuscript Title: : "Long-Term Anifrolumab Treatment Normalizes Hematologic Parameters and Several Serologic Markers in Patients With Systemic Lupus Erythematosus".

Manuscript Number (if known): Click or tap here to enter text.

In the interest of transparency, we ask you to disclose all relationships/activities/interests listed below that are related to the content of your manuscript. "Related" means any relation with for-profit or not-for-profit third parties whose interests may be affected by the content of the manuscript. Disclosure represents a commitment to transparency and does not necessarily indicate a bias. If you are in doubt about whether to list a relationship/activity/interest, it is preferable that you do so.

The author's relationships/activities/interests should be defined broadly. For example, if your manuscript pertains to the epidemiology of hypertension, you should declare all relationships with manufacturers of antihypertensive medication, even if that medication is not mentioned in the manuscript.

In item #1 below, report all support for the work reported in this manuscript without time limit. For all other items, the time frame for disclosure is the past 36 months.

		**Name all entities with whom you have this relationship or indicate none (add rows as needed)**	**Specifications/Comments (e.g., if payments were made to you or to your institution)**
**Time frame: Since the initial planning of the work**			
1	All support for the present manuscript (e.g., funding, provision of study materials, medical writing, article processing charges, etc.) **No time limit for this item.**	☐ None	
			I´m an AstraZeneca employee.
			Click the tab key to add additional rows.
**Time frame: past 36 months**			
**2**	Grants or contracts from any entity (if not indicated in item #1 above).	☒ **None**	
**3**	Royalties or licenses	☒ **None**	

		Name all entities with whom you have this relationship or indicate none (add rows as needed)	Specifications/Comments (e.g., if payments were made to you or to your institution)
**4**	Consulting fees	☒ **None**	
**5**	Payment or honoraria for lectures, presentations, speakers bureaus, manuscript writing or educational events	☒ **None**	
**6**	Payment for expert testimony	☒ **None**	
**7**	Support for attending meetings and/or travel	☒ **None**	
**8**	Patents planned, issued or pending	☐ **None**	
			AZ patents.
**9**	Participation on a Data Safety Monitoring Board or Advisory Board	☒ **None**	
**10**	Leadership or fiduciary role in other board, society, committee or advocacy group, paid or unpaid	☒ **None**	

		Name all entities with whom you have this relationship or indicate none (add rows as needed)	Specifications/Comments (e.g., if payments were made to you or to your institution)
**11**	Stock or stock options	☐ **None**	
		AstraZeneca	
**12**	Receipt of equipment, materials, drugs, medical writing, gifts or other services	☒ **None**	
**13**	Other financial or non-financial interests	☐ **None**	
			I´m an AstraZeneca employee.

Please place an "X" next to the following statement to indicate your agreement:

☒ I certify that I have answered every question and have not altered the wording of any of the questions on this form.
