# Supplementary material for: Long‐Term Anifrolumab Treatment Normalizes Hematologic Parameters and Several Serologic Markers in Patients With Systemic Lupus Erythematosus
Source: ACR Open Rheumatol. 2026 Jun 15;8(6):e90065. doi: 10.1002/acr2.90065 (PMC13267668; doi:10.1002/acr2.90065)
Supplement: Supplementary file 2 — Data S1. Supporting Information. [file ACR2-8-e90065-s002.docx]

SUPPLEMENTAL MATERIAL

**Long-Term Anifrolumab Treatment Normalizes Hematologic Parameters and Several Serologic Markers in Patients With Systemic Lupus Erythematosus**

**Running head: Long term anifrolumab and hematologic SLE**

Edward M. Vital, PhD, FRCP,^1^ Zahir Amoura, MD, MSc,^2^ Kenneth C. Kalunian, MD,^3^ Ian N. Bruce, MD,^4,5^ Yoshiya Tanaka MD, PhD,^6^ Susan Manzi, MD, MPH,^7^ Ihor Hupka, MD,^8^ Jacob Knagenhjelm,^9^ Hussein Al-Mossawi MD, PhD, MRCP,^10^ and Catharina Lindholm, MD PhD^9^

^1^Leeds Institute of Rheumatic and Musculoskeletal Medicine, Faculty of Medicine and Health, University of Leeds, Leeds, United Kingdom

^2^French National Reference Center for SLE, Hôpital La Pitié-Salpêtrière, Sorbonne University, Paris, France

^3^University of California San Diego School of Medicine, La Jolla, California, United States of America

^4^Centre for Musculoskeletal Research, The University of Manchester, Manchester, United Kingdom

^5^Centre for Public Health, Faculty of Medicine, Health and Life Sciences, Queen's University, Belfast, Belfast, United Kingdom

^6^The First Department of Internal Medicine, University of Occupational and Environmental Health, Japan, Kitakyushu, Japan

^7^Lupus Center of Excellence, Autoimmunity Institute, Allegheny Health Network, Pittsburgh, Pennsylvania, United States of America

^8^BioPharmaceuticals R&D, AstraZeneca, Warsaw, Poland

^9^BioPharmaceuticals R&D, AstraZeneca, Gothenburg, Sweden

^10^St Edmund Hall, University of Oxford, Oxford, United Kingdom

**Corresponding author:**Edward M. Vital

ORCID: 0000-0003-1637-4755

Leeds Institute of Rheumatic and Musculoskeletal Medicine

Faculty of Medicine and Health, University of Leeds

2nd Floor, Chapel Allerton Hospital, Chapeltown Road

Leeds, LS7 4SA

United Kingdom

**Phone:** +44(0)113 392 4879

**Email:** [E.M.J.Vital@leeds.ac.uk](mailto:E.M.J.Vital@leeds.ac.uk)

**Fax:** +44 (0) 113 392 4991

**FUNDING**

This study and medical writing support was funded by AstraZeneca. The study sponsor was involved in the study design, data collection, analysis, and interpretation, and in the decision to submit the paper for publication.

Supplementary Table S1. Hematologic and serologic parameters at TULIP baseline in the LTE population.

| **Characteristic** | | **Anifrolumab 300 mg**  **(n = 257)** | **Placebo**  **(n = 112)** |
| --- | --- | --- | --- |
| **Hematology parameters, n (%) unless otherwise stated** | | | |
| **Lymphocytes** | Mean (± SD), GI/L | 1.3 (0.7) | 1.4 (0.6) |
|  | Low (< 1 GI/L) | 100 (38.9) | 40 (35.7) |
|  | Normal (≥ 1 GI/L and < 4 GI/L) | 156 (60.7) | 71 (63.4) |
|  | High (≥ 4 GI/L) | 1 (0.4) | 1 (0.9) |
| **Hemoglobin** | Mean (± SD), g/L | 125.7 (14.9) | 128.7 (14.8) |
|  | Low (< 120 g/L) | 84 (32.7) | 29 (25.9) |
|  | Normal/High (≥ 120 g/L) | 173 (67.3) | 74.1 (4.1) |
| **Platelets** | Mean (± SD), GI/L | 238.7 (74.0) | 249.0 (74.1) |
|  | Low (< 150 GI/L) | 24 (9.3) | 11 (9.8) |
|  | Normal (≥ 150 and < 450 GI/L) | 229 (89.1) | 100 (89.3) |
|  | High (≥ 450 GI/L) | 4 (1.6) | 1 (0.9) |
| **Neutrophils** | Mean (± SD), GI/L | 3.8 (1.9) | 4.1 (2.0) |
|  | Low (< 1.5 GI/L) | 9 (3.5) | 4 (3.6) |
|  | Normal/High (≥ 1.5 GI/L) | 248 (96.5) | 108 (96.4) |
| **Serology markers^a^** | | | |
| **IgG** | Mean (± SD), g/L | 13.9 (5.0) | 13.9 (5.0) |
| **IgA** | Mean (± SD), g/L | 3.0 (1.7) | 3.1 (1.3) |
| **IgM** | Mean (± SD), g/L | 1.1 (0.8) | 1.0 (0.7) |

^a^Mean (± SD) calculated from n = 253 patients in the anifrolumab group and n = 112 patients in the placebo group.

Ig, immunoglobulin; LTE, long-term extension; SD, standard deviation.

Supplementary Figure S1. Effect of anifrolumab treatment on lymphocyte levels in the LTE population. (A) Change from baseline in lymphocyte levels. (B) Proportions of patients with low or normalized lymphocyte levels over time in patients with lymphopenia at baseline.^a,b^


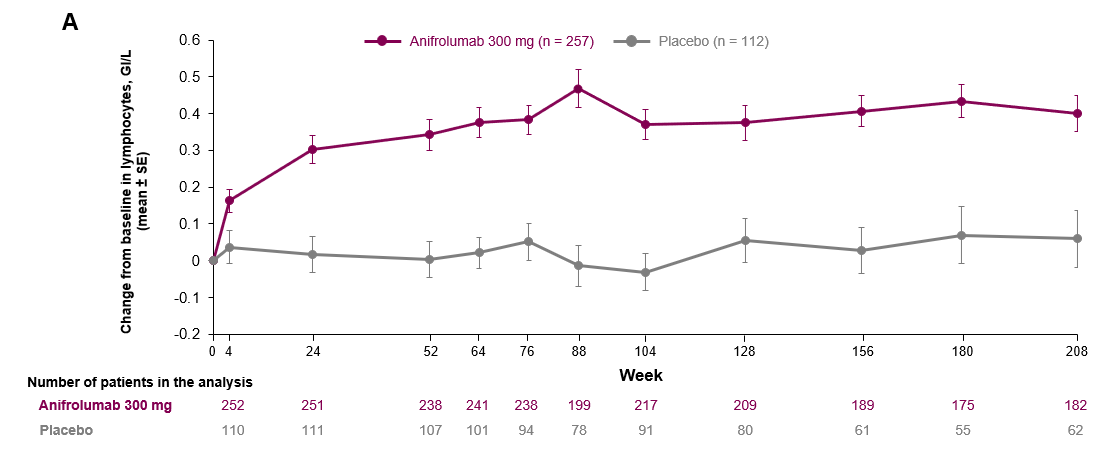


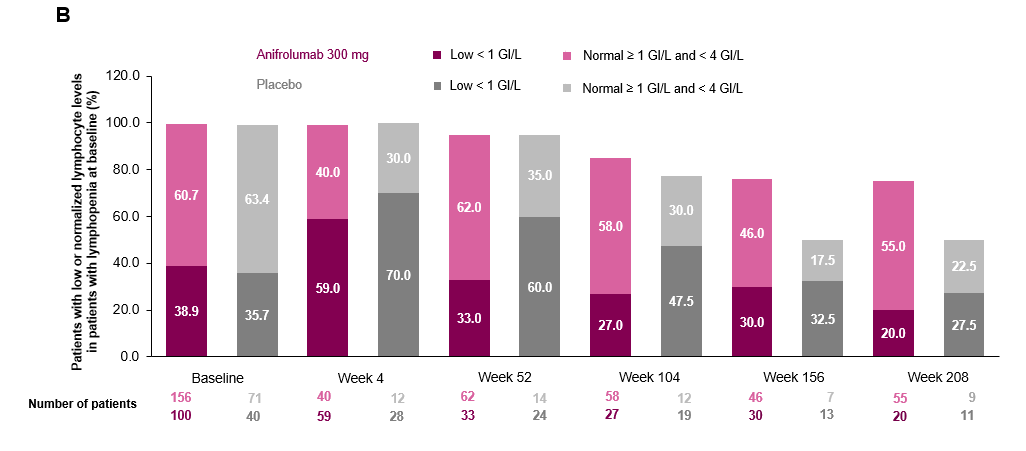


^a^The proportions of patients in panel B do not add up to 100% due to missing data. ^b^Baseline data only shows proportions of patients with lymphopenia (low) or normal lymphocyte levels at baseline. Data for patients with high lymphocyte levels are not shown in this analysis.

LTE, long-term extension; SE, standard error.

Supplementary Figure S2. Withdrawal rates over time in patients with lymphopenia at baseline in the combined TULIP + LTE population.


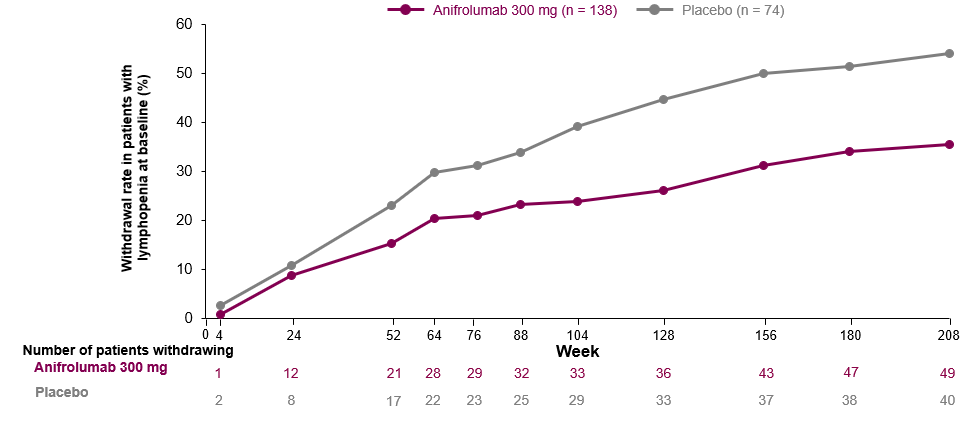


Supplementary Figure S3. Effect of anifrolumab treatment on hemoglobin levels in the LTE population. (A) Change from baseline in hemoglobin levels. (B) Hemoglobin normalization over time in patients with anemia at baseline.


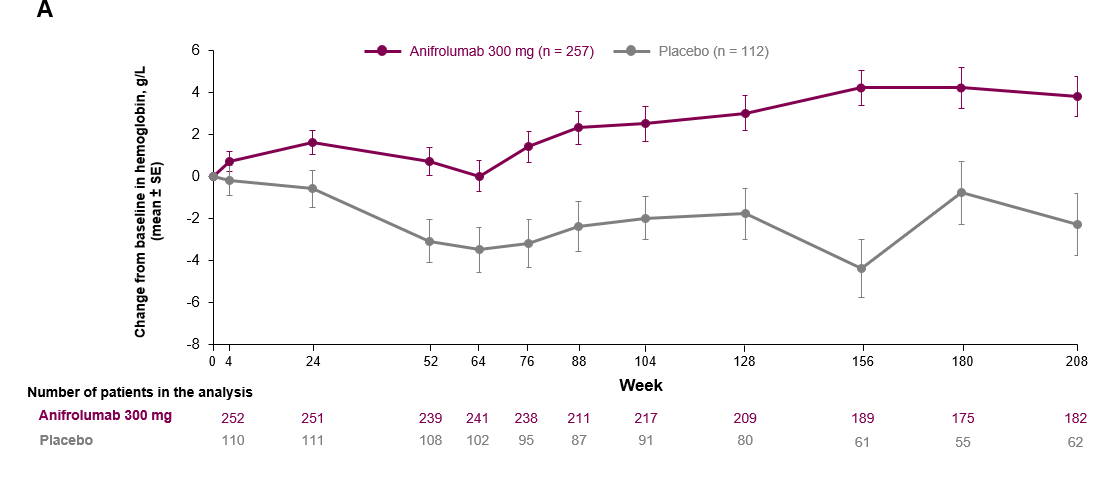


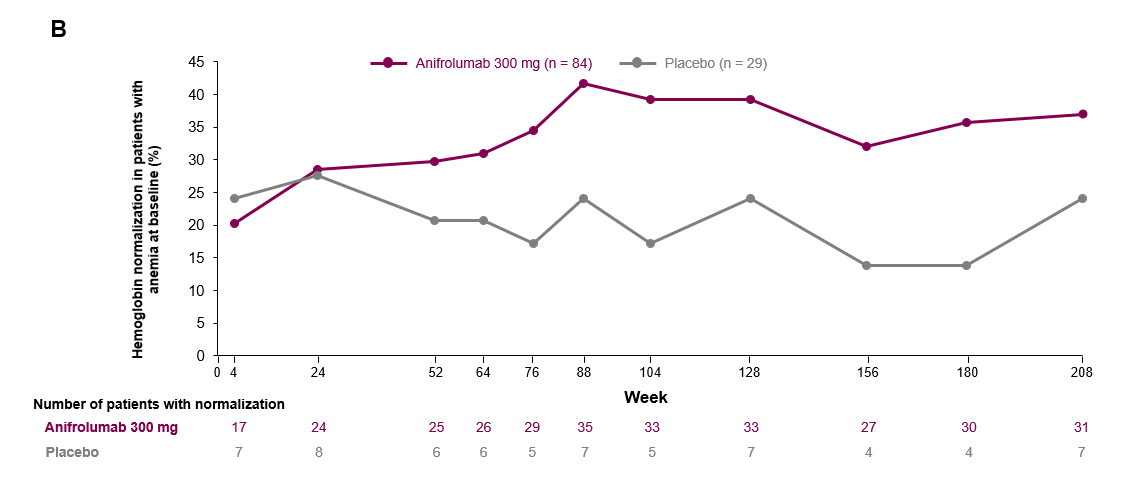


LTE, long-term extension; SE, standard error.

Supplementary Figure S4. Withdrawal rates over time in patients with anemia at baseline in the combined TULIP + LTE population.


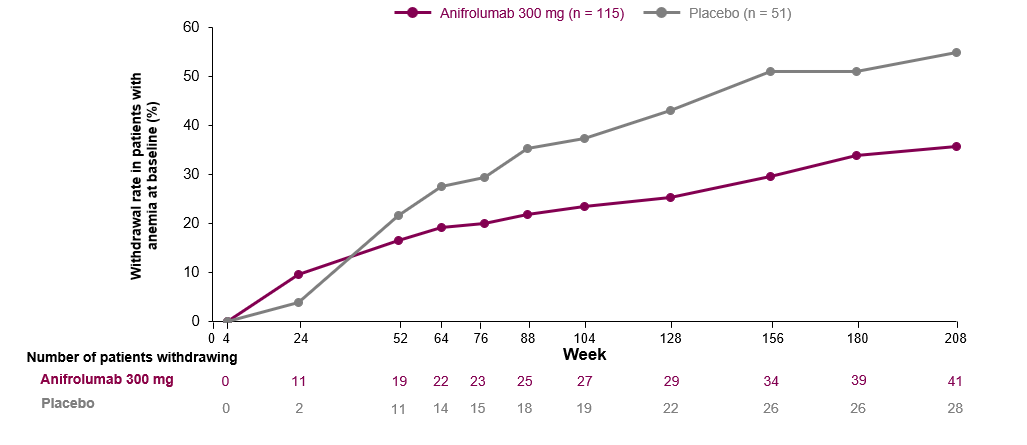


Supplementary Figure S5. Effect of anifrolumab treatment on platelet levels in the LTE population. (A) Change from baseline in platelet levels.^a^ (B) Platelet normalization over time in patients with thrombocytopenia at baseline.


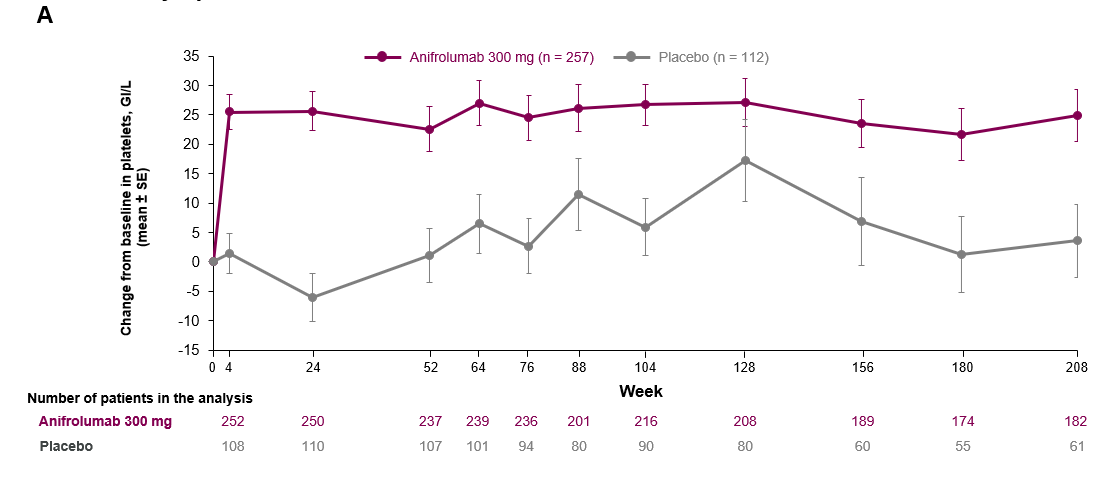


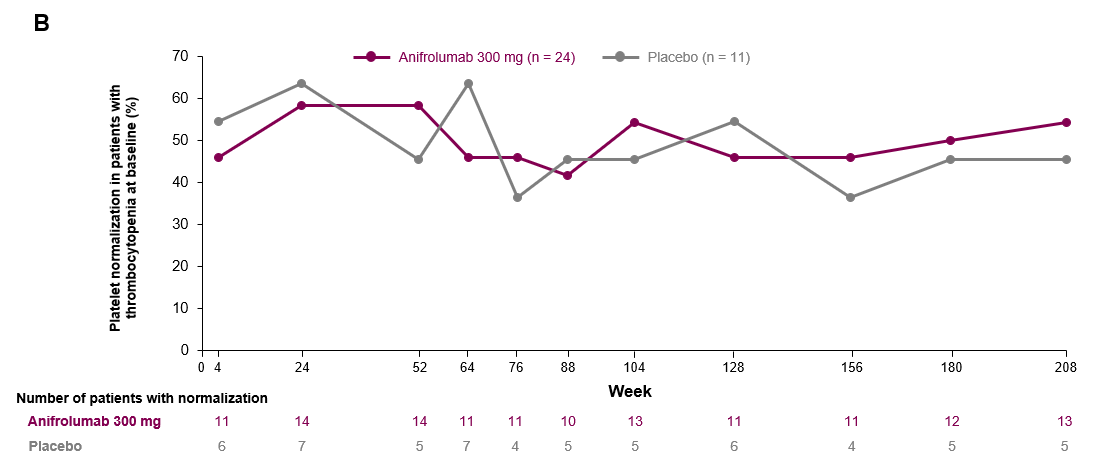


^a^No increases in mean platelet levels from baseline led to high platelet counts above the normal range at any timepoint (i.e., mean levels did not exceed ≥450 GI/L).

Long-term extension; SE, standard error.

Supplementary Figure S6. Withdrawal rates over time in patients with thrombocytopenia at baseline in the combined TULIP + LTE population.


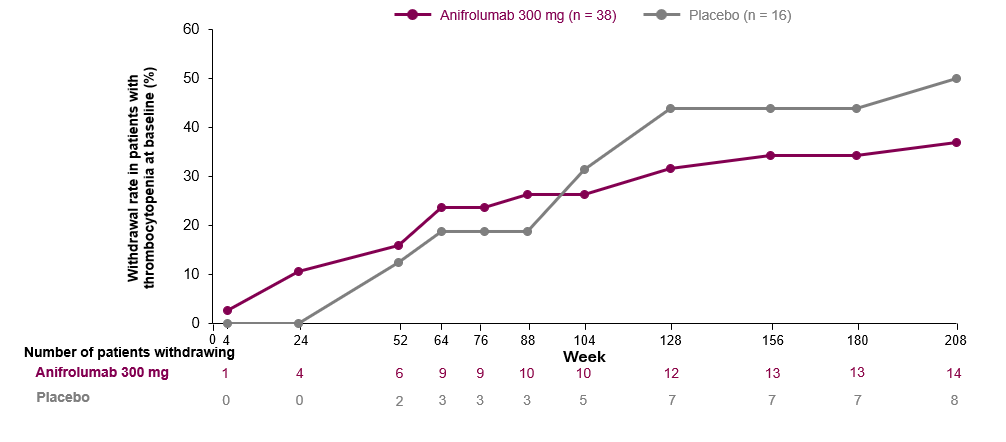


Supplementary Figure S7. Effect of anifrolumab treatment on BICLA response by baseline lymphocyte, hemoglobin, and platelet levels in the combined TULIP + LTE population.


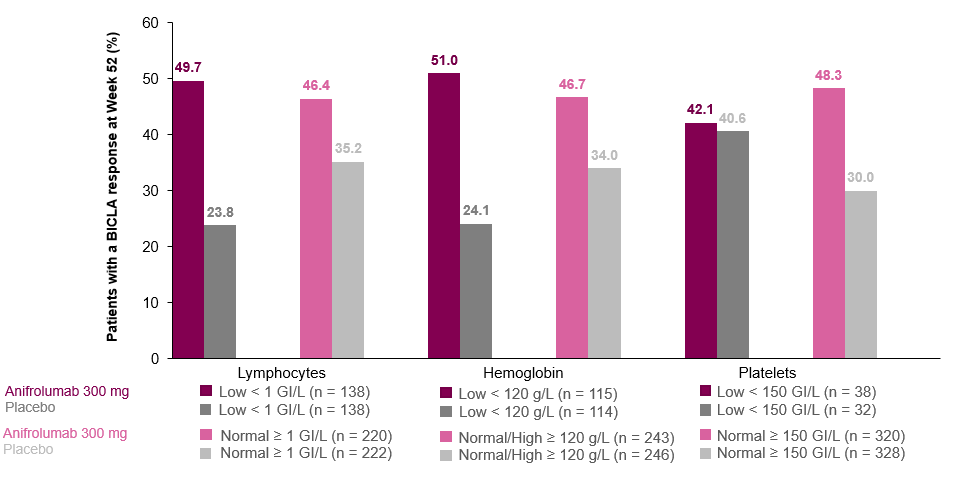


BICLA, British Isles Lupus Assessment Group (BILAG)-based Composite Lupus Assessment.

Supplementary Figure S8. Effect of anifrolumab treatment on neutrophil levels in the combined TULIP + LTE population. (A) Change from baseline in neutrophil levels over time. (B) Change from baseline in neutrophils over time in patients with neutropenia at baseline (< 1.5 GI/L).^a^


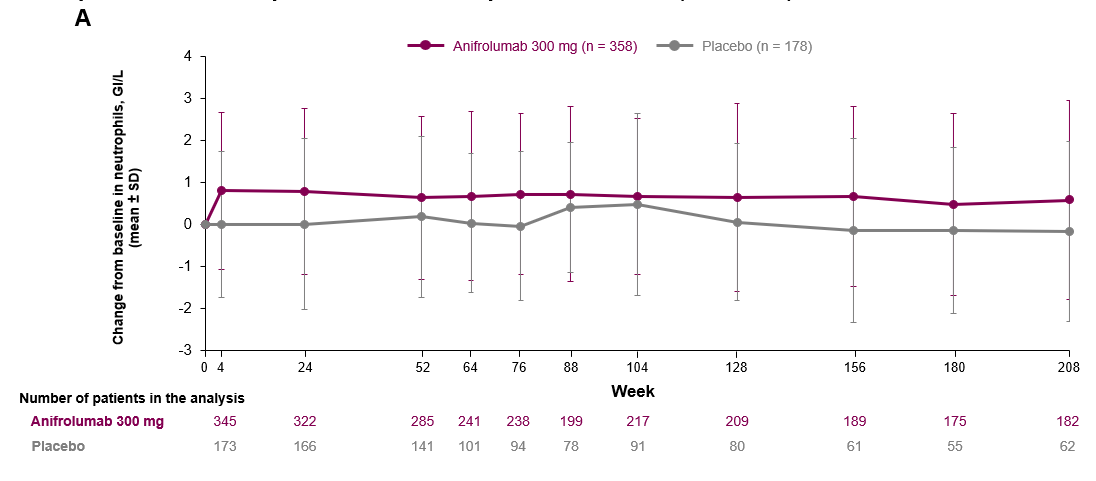


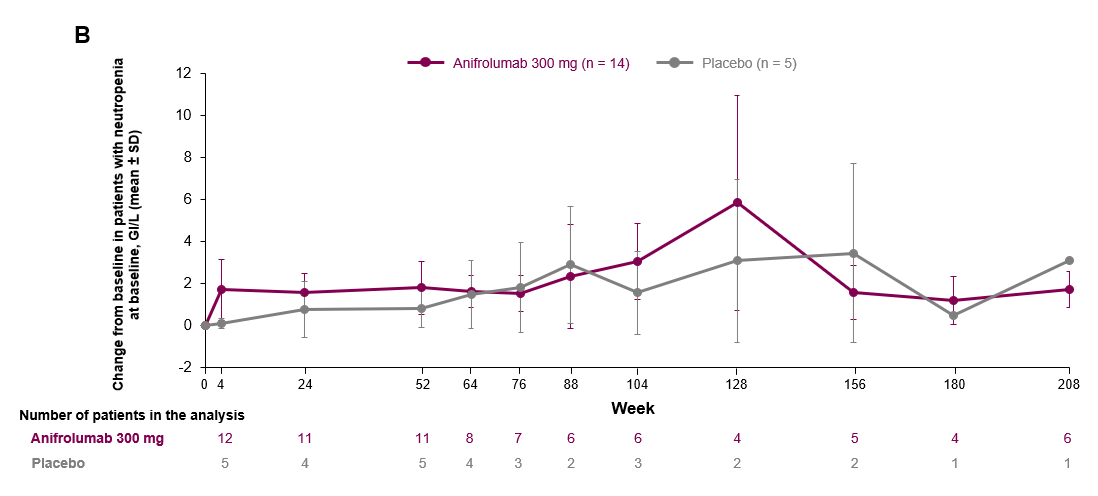


^a^SDs could not be calculated for the placebo group at Weeks 180 or 208 (n = 1).

SD, standard deviation.

Supplementary Figure S9. Effect of anifrolumab treatment on neutrophil levels in the LTE population.. (A) Change from baseline in neutrophil levels over time. (B) Change from baseline in neutrophils over time in patients with neutropenia at baseline (< 1.5 GI/L).^a^


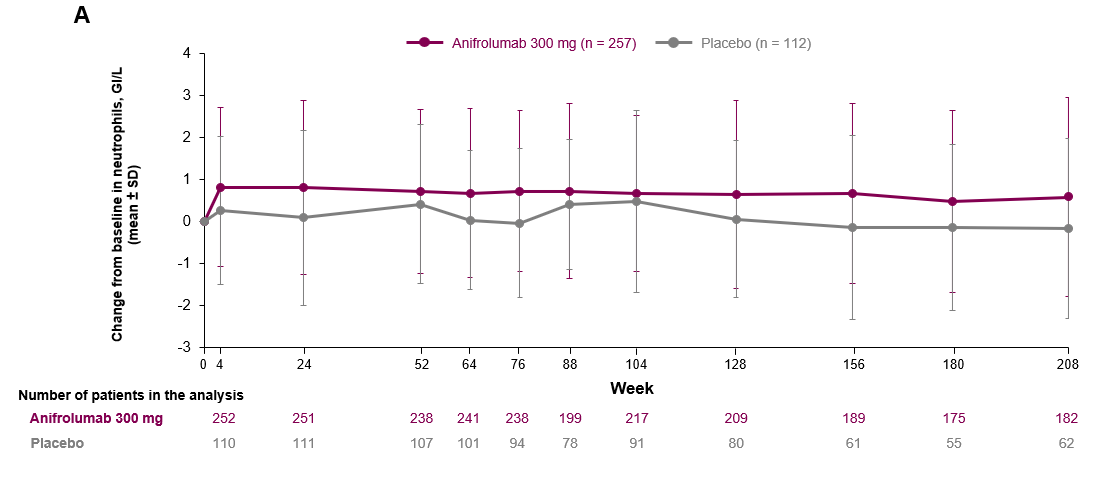


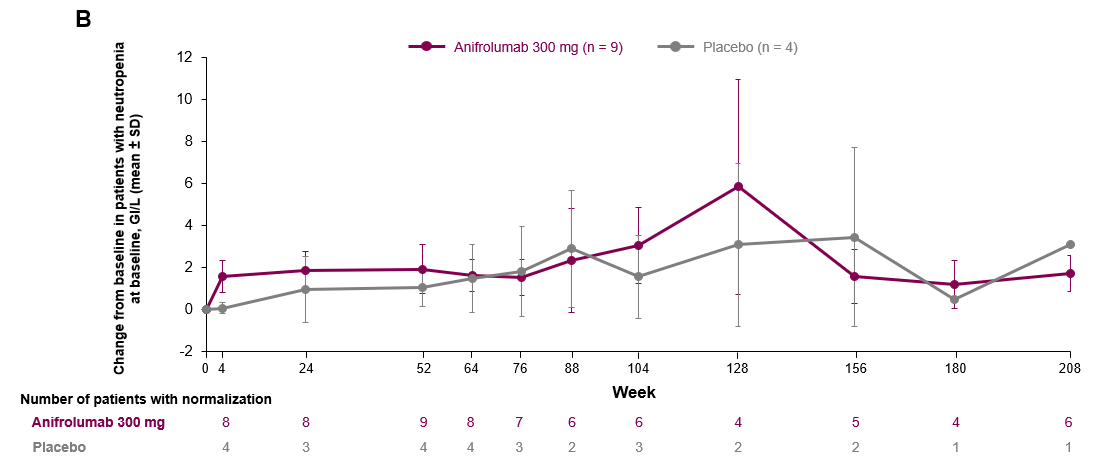


^a^SDs could not be calculated for the placebo group at Weeks 180 or 208 (n = 1).

SD, standard deviation.

Supplementary Figure S10. Effects of anifrolumab treatment on serologic markers in the LTE population. Change from baseline in levels of (A) anti‑dsDNA, (B) C3, and (C) C4 over time among patients who were anti-dsDNA positive or had low C3 or C4 at baseline, respectively.


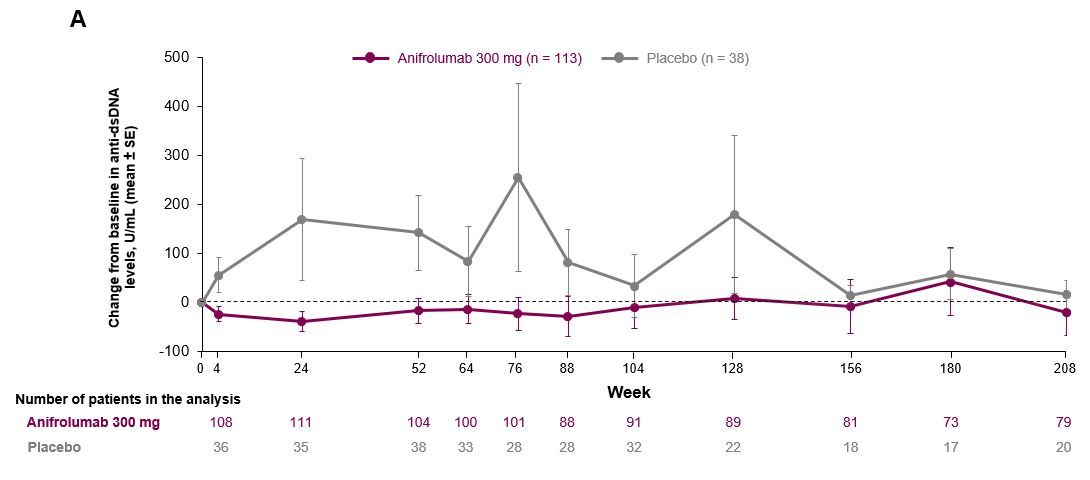


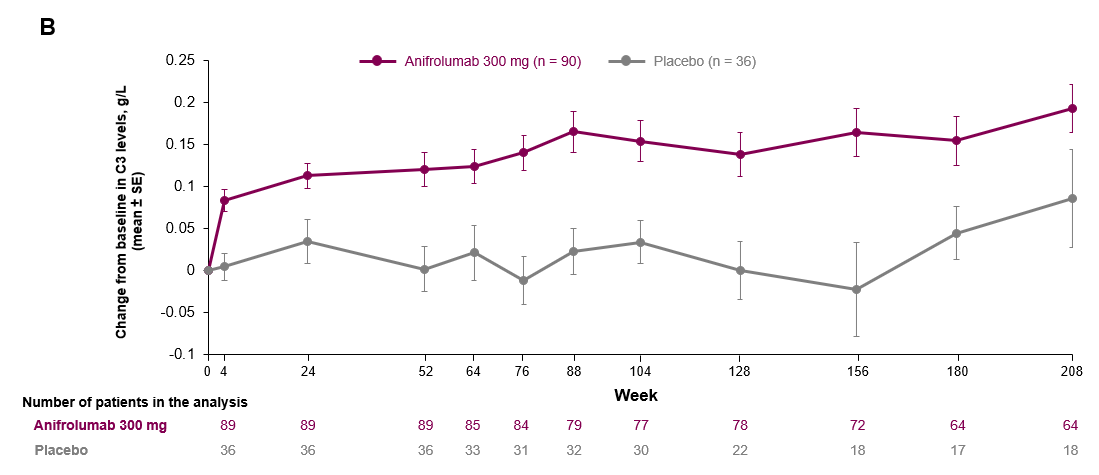


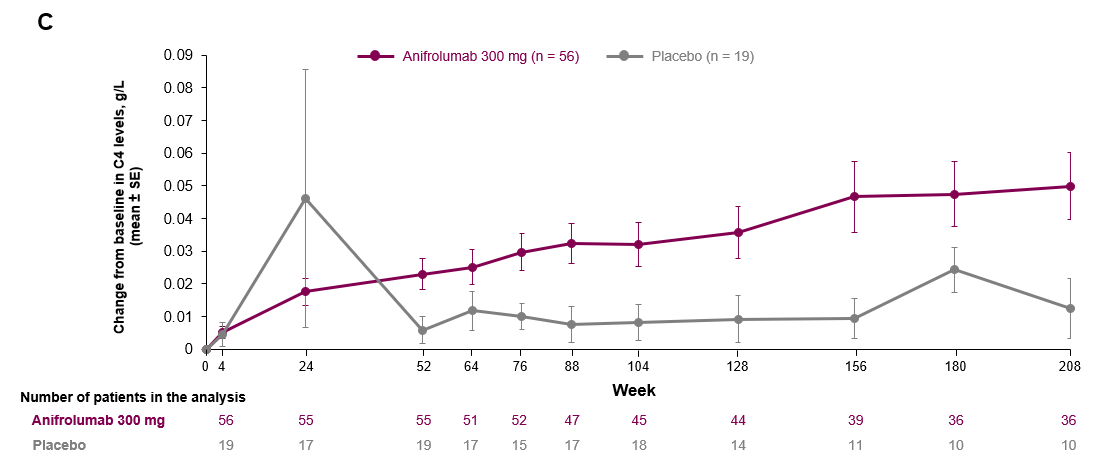


C3, complement 3; C4, complement 4; dsDNA, double-stranded DNA; LTE, long-term extension; SE, standard error.
